# Uptake of ^14^CO_3_^2−^ by HCP (CEM III/C and CEM III/C + CaCO_3_) in the presence and absence of ISA and chloride

**DOI:** 10.1039/d6ra04948f

**Published:** 2026-08-21

**Authors:** Rosa Ester Guidone, Nils Huber, Barbara Lothenbach, Florian Bocchese, Stéphane Brassinnes, Marcus Altmaier, Xavier Gaona

**Affiliations:** a Karlsruhe Institute of Technology (KIT), Institute for Nuclear Waste Disposal (INE) Eggenstein-Leopoldshafen Germany rosa.guidone@kit.edu xavier.gaona@kit.edu; b Laboratory for Concrete & Asphalt, Empa, Swiss Federal Laboratories for Materials Science and Technology Dübendorf Switzerland; c ONDRAF/NIRAS, Belgian Agency for Radioactive Waste and Enriched Fissile Materials Brussels Belgium

## Abstract

The retention of ^14^CO_3_^2−^ by hydrated cement pastes (CEM III/C; CEM III/C + CaCO_3_) was investigated in the absence and presence of isosaccharinate (ISA, 10^−5^–0.2 M) and NaCl (≈10^−3^–2.0 M). The moderate retention observed for both materials at short contact times (*t* = 2 days) is primarily attributed to adsorption on C–S–H phases. The differential retention behaviour at longer contact times is tentatively explained by the different assembly of carbonate-bearing phases (calcite, monocarbonate, hydrotalcite) in both binders, which are responsible for isotopic exchange processes. ISA has a minor impact on ^14^CO_3_^2−^ retention except at high [ISA] and low S/L ratios, where partial cement dissolution is triggered by the formation of CaISA_-H_(aq) and Ca(ISA)_2_(cr). NaCl has a negligible impact on the retention of ^14^CO_3_^2−^, in spite of its influence on surface charge and phase distribution. This work provides an improved quantitative description and mechanistic understanding of ^14^CO_3_^2−^ retention in cementitious systems.

## Abstract 2

Assessment of ^14^CO_3_^2−^ retention by cement-based materials in the presence and absence of ISA and NaCl, under chemical conditions relevant for the near-surface repository of radioactive waste.

## Graphical abstract

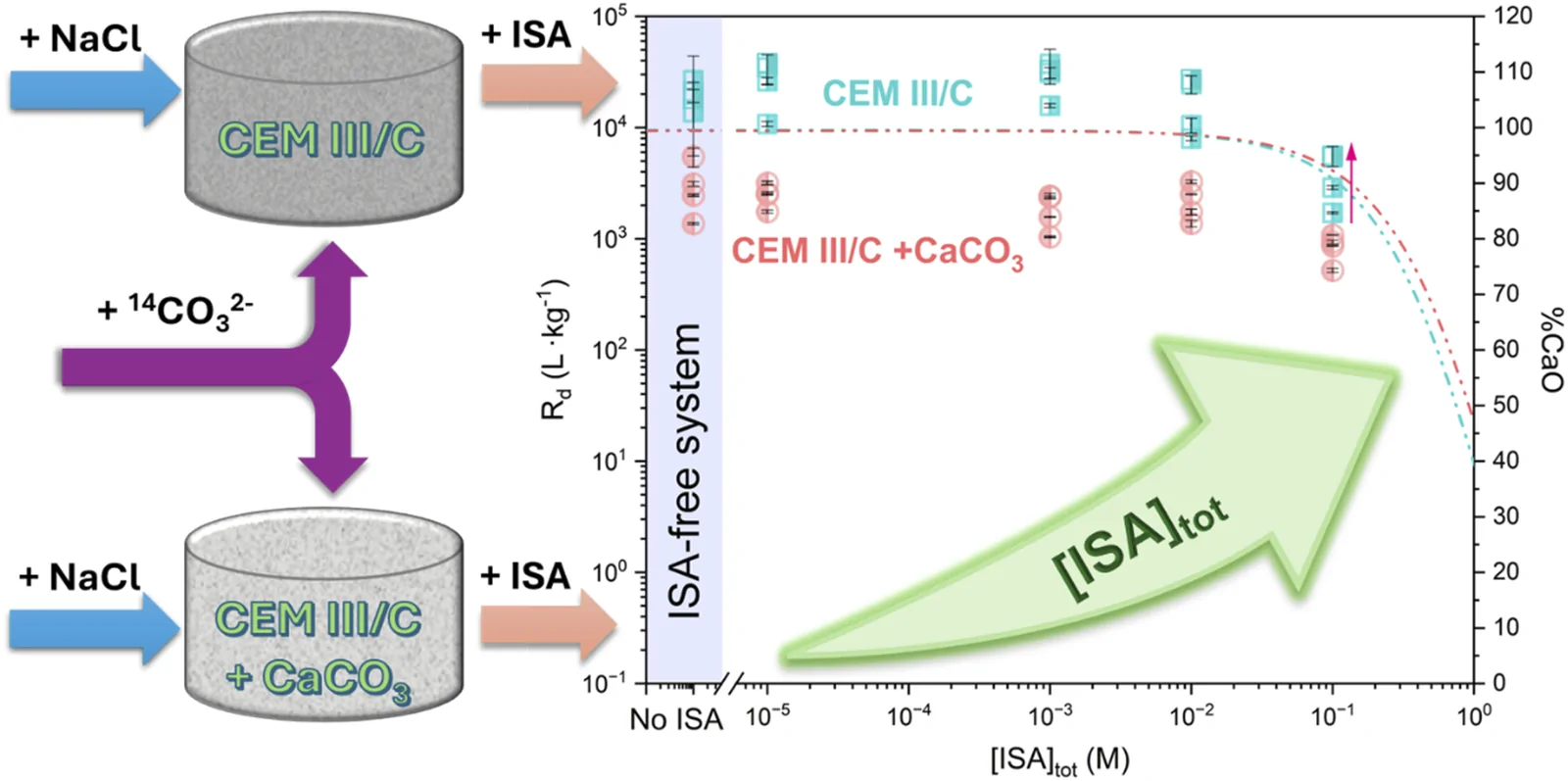

## 1 Introduction

The production of radiocarbon or carbon-14 (^14^C) occurs in nuclear power plants by activation of stable parent isotopes (^14^N, ^13^C, ^17^O) found, for example, in core structural materials, reactor coolants and fuel impurities.^1,2^ Although the overall ^14^C inventory in radioactive waste can include both organic and inorganic forms, the latter is expected to prevail in the context of low- and intermediate level short-lived waste (L/ILW-SL).^1^

Cementitious materials are extensively used for the construction of engineered barrier systems and stabilization/immobilization of radioactive waste, mostly (but not exclusively) in the context of near-surface and underground repositories for L/ILW. Beyond their structural properties, cementitious materials also exhibit excellent retention properties towards radionuclides and other contaminants present in the waste.^1,3^ The mineralogical composition of hydrated cements is generally constituted by C–S–H (calcium–silicate–hydrates) phases (*x*CaO·SiO_2_·*y*H_2_O with approx. 0.6 < *x* < 1.8), portlandite (Ca(OH)_2_), AFm (aluminoferrite monosulfate) phases (with a general formula Ca_3_(Al,Fe)(OH)_6_·[12H_2_O]X_3_·*n*H_2_O, with X = M^2+^ or 2M^+^), ettringite (Ca_6_Al_2_(SO_4_)_3_(OH)_12_·26H_2_O or Ca_6_Fe_2_(SO_4_)_3_(OH)_12_·26H_2_O) as a member of the AFt (aluminoferrite trisulfate) phases, hydrogarnet (3CaO·Al_2_O_3_·6H_2_O) and hydrotalcite (Mg_6_Al_2_CO_3_(OH)_16_·4(H_2_O)) as secondary reaction product.^4^ C–S–H phases, as the main component of hydrated cement paste (HCP), are defined by a poorly crystalline structure and high surface area (≈150 m^2^ g^−1^).^5^ Surface properties of C–S–H phases such as the surface charge strongly depend on the Ca^2+^ concentration and pH of the pore solution.^6,7^ By means of zeta potential measurements, Pointeau *et al.* reported an increase of the surface charge of OPC (ordinary Portland cement) from −17 mV to +20 mV, when the Ca concentrations increased from 6 to 22 mM.^8^ AFm phases (*e.g.* monocarbonate or monosulfonate) and hydrotalcite-like phases are characterized by a layered structure, where the positive charge of the octhahedral layers, [Ca_2_(Al,Fe)(OH)_6_·2H_2_O]^+^ (AFm phases) and [M^II^_1−*x*_M^III^_*x*_(OH)_2_]^*x*+^ (in the case of hydrotalcite, *x* = 0.25 and M^II^:M^III^ = 3) is compensated by anions bound in the interlayer region between two adjacent layers. Due to these structural features, AFm phases and hydrotalcite show a high affinity to bind anionic species, *e.g.*, Cl^−^,^9,10^ I^−^,^11^ SeO_4_^2−^ (ref. 12) or MoO_4_^2−^.^13^

Supplementary cementitious materials (SCM), such as blast-furnace slag (BFS) and ground granulated blast-furnace slag (GGBFS), are mixed in concrete or blended cements in order to minimize the CO_2_ footprint mainly associated with the production of clinker.^14^ GGBFS, a by-product of pig iron production, is obtained by rapid cooling of molten slag collected at the top of molten iron and grinded into a fine powder before further use. Due to the rapid quenching, GGBFS is mainly composed of a glassy phase, which is characterized by higher content of SiO_2_ and Al_2_O_3_ compared to OPC. GGBFS can be used as a direct replacement of OPC, and replacement rates may vary from 36% up to 95%.^15^ High replacements level of GGBFS, as in the case of CEM III/C cements, improve the durability of concrete in aggressive environments (*e.g.*, high sulphate and chloride concentrations), due to the much lower permeability, and reduce the heat of hydration.^16–18^ Blending OPC with GGBFS results in the formation of C–S–H phases with lower Ca/Si ratio whilst decreasing portlandite content. The presence of C–S–H, AFm phases, ettringite, and hydrotalcite-like phases has been observed experimentally for OPC/GGBFS blended systems.^19–23^ Although the higher alumina (Al_2_O_3_) content present in the GGBFS could lead to the formation of higher fractions of AFm and AFt, experimental studies with OPC/BFS (or GGBFS) blended cements reported instead lower fractions of such phases^21,22^ and a redistribution of Al involving also the incorporation in the C–S–H phases, *i.e.*, formation of C–A–S–H phases.^22,24–26^ Given the slow hydration kinetics of GGBS,^27^ the fraction of C–A–S–H phases can be lower than in PC, but is expected to increase over time.

The addition of low amounts of limestone (CaCO_3_) in OPC has been found to increase the formation of monocarbonate and hemicarbonate and stabilize ettringite.^28–30^ The stabilization of ettringite and monocarbonate in the presence of limestone contributes to an increase of total volume of cement phases which results in higher compressive strength. Such effects have been also observed for OPC/BFS blended cements in the presence of limestone.^31^ The presence of limestone lowers the Al concentration in cement pore solution, in agreement with the precipitation of more ettringite and monocarbonate. This observation is also consistent with the lower Al/Si observed in C–S–H phases and higher Mg/Al ratio observed for hydrotalcite-like phases in ternary blend systems.^31^

The retention mechanism of inorganic ^14^C by hydrated cement paste is expected to occur *via* two main sorption mechanisms: (i) electrostatic adsorption between the negatively charged ^14^CO_3_^2−^ and the positively charged surfaces of C–S–H phases with high Ca/Si ratio,^32–34^ and (ii) isotopic exchange of ^14^CO_3_^2−^ with the natural carbonate groups of calcite and other carbonate-bearing phases such as monocarbonate in hydrated cement. The latter process is expected to occur through recrystallization processes, eventually involving slow kinetics.^35,36^ The surface adsorption of ^14^CO_3_^2−^ on C–S–H phases (Ca/Si = 0.83–1.5) was investigated by Noshita and co-authors.^32^ Based on the observed decrease in the retention with decreasing Ca/Si ratio, the authors concluded that ^14^CO_3_^2−^ is possibly adsorbing on positively charged surface sites prevailing at high Ca/Si ratios (*i.e.*, –SiOCa^+^) *via* electrostatic interactions. The role of surface charge of C–S–H phases towards the uptake of anionic species was also pointed out in the sorption experiments carried out by Pointeau *et al.* with HCP (CEM I) at 11.5 ≤ pH ≤ 13.3.^34^ In our recent study on the retention of ^14^CO_3_^2−^ by HCP (CEM I),^37^ we observed that both isotopic exchange with carbonate-containing phases (*e.g.* monocarbonate, calcite) and adsorption on C–S–H phases play a relevant role in the overall retention of ^14^CO_3_^2−^. By means of sorption and diffusion experiments, Rasamimanana *et al.* observed steady conditions (*R*_d_ ≈ 10^3^ L kg^−1^) for the retention of ^14^CO_3_^2−^ by CEM V/A (pH = 13.5) after a contact time of 30 days.^38^ CEM V/A mixture also contains blast furnace glass but in minor concentration (18–30%) compared to CEM III/C, together with pozzolana or fly ash (18–30%) and Portland cement clinker (40–64%).^15^ The authors^38^ underlined the presence of two sorption regimes as function of the equilibration time: surface adsorption onto C–S–H phases and portlandite at short contacting time, and isotopic exchange with carbonate phases of cement at long contacting times. Bayliss and co-authors studied the retention of ^14^CO_3_^2−^ by two different cementitious binders (Sulfate Resistant Portland Cement, SRPC and OPC/BFS) under the variation of the solid-to-liquid ratio (10 g L^−1^ ≤ S/L ≤ 100 g L^−1^) and initial radiotracer concentration (1.5 × 10^−9^ M ≤ [^14^CO_3_^2−^]_tot_ ≤ 9.5 × 10^−7^ M).^36^ The authors observed the increase of distribution ratios (*R*_d_) with increasing contact time, [^14^CO_3_^2−^]_tot_ and S/L, and concluded that uptake was mainly driven by isotopic exchange. Bradbury and Sarott were able to reproduce the *R*_d_ values reported by Bayliss *et al.* for the BFS/OPC system, by introducing a new empirical evaluation of the distribution coefficients.^39^ The authors proposed that the prevailing mechanism responsible for the uptake of ^14^CO_3_^2−^ at long contacting times is isotopic exchange with the fraction of calcite present in cement. The authors accordingly proposed that the retention of ^14^CO_3_^2−^ by cement is independent of [^14^CO_3_^2−^]_tot_ and can be evaluated by means of the effective sorption parameter calculated as the ratio of total inventory of calcite present in the solid, and the solubility limit of calcite in the pore water. Bradbury and Sarott introduced also the so-called accessibility factor (*α*), which takes into account the fraction of carbonate-containing phases accessible to the isotopic exchange.

Large inventories of cellulose-based materials (*e.g.*, tissue, cotton, wood) are expected in repositories for L/ILW-SL. In the hyperalkaline conditions defined by cementitious systems (pH ≥ 10), cellulose undergoes degradation, resulting in the formation of isosaccharinic acid (ISA, C_6_H_12_O_6_) as main degradation product.^40–42^ ISA has been reported to form strong complexes with hard Lewis acids (*e.g.*, Pu(iii/iv),^43–45^ Zr(iv),^46^ Th(iv),^47,48^*etc.*) under alkaline conditions, but also to compete with anionic species (*e.g.*, ^75^SeO_3_^2−^) for sorption sites on the cement surface.^34^ To date, very few studies have been conducted on the potential impact of ISA on the retention of ^14^CO_3_^2−^ by HCP. In our recent study targeting the CEM I-^14^CO_3_^2−^-ISA system,^37^ we observed that the retention of ^14^CO_3_^2−^ is not affected by the presence of ISA up to ISA concentrations ≤ 10^−2^ M. The slight decrease in *R*_d_ values observed at higher ISA concentrations and low solid-to-liquid (S/L) ratios was explained by the partial dissolution of cement triggered by the formation of Ca-ISA aqueous complexes and precipitation of Ca(ISA)_2_(cr), as also supported by thermodynamic calculations determined considering ISA in equilibrium with portlandite. Similar observations were reported for the uptake of H^14^CO_3_^2−^/^14^CO_3_^2−^ by calcite under weakly alkaline conditions (pH ≈ 8.3).^49^

High inventories of stable chloride arising from specific waste streams can influence the mineral composition of cementitious materials, and may thus affect the retention of anionic species such as ^14^CO_3_^2−^.^1^ Possible competition effects between Cl^−^ and CO_3_^2−^ for the positive surface sites of cement may also contribute to decrease ^14^CO_3_^2−^ retention. In the case of CEM I, the formation of Friedel's salt together with the destabilization of monocarbonate observed at high chloride concentration, resulted in a minor impact in the retention mechanism of ^14^CO_3_^2−^, as quantified for both (cement-NaCl–^14^CO_3_^2−^) ternary and (cement-NaCl–^14^CO_3_^2−^-ISA) quaternary systems.^37^

Following our previous studies with calcite^49^ and CEM I,^37^ the present work focuses on the uptake of ^14^CO_3_^2−^ by HCP with high slag content (∼84%, CEM III/C)^50^ in the absence and presence of limestone (CaCO_3_). The impact of ISA and chloride (as NaCl) on the retention of ^14^CO_3_^2−^ is investigated as well by means of systematic batch sorption experiments on the ternary (cement-^14^CO_3_^2−^-ISA, cement-NaCl–^14^CO_3_^2−^) and quaternary (cement-NaCl–^14^CO_3_^2−^-ISA) systems.

## 2 Experimental

### 2.1 Chemicals and analytical methods

All samples were prepared and stored under Ar-atmosphere (O_2_ < 1 ppm) at *T* = (22 ± 2) °C. Solution preparation was carried out with ultra-pure water (Millipore, 18.2 MΩ cm) purged with Ar for several hours (*t* > 3 hours) in order to remove dissolved CO_2_(g).

Sorption experiments were performed using ^14^CO_3_^2−^ as radiotracer. The ^14^CO_3_^2−^ (*t*_1/2_ = 5700 a) stock solution was purchased from Eckert & Ziegler Nuclitec GmbH with a purity of > 99.9%. The matrix composition of ^14^CO_3_^2−^ stock solution consisted of 0.1 M NaOH, containing as inactive carrier [Na_2_CO_3_] = 2.8 × 10^−4^ M. The radiotracer stock solution was diluted in cement pore water solution (see Section 2.2) to achieve [^14^CO_3_^2−^] = 4.3 × 10^−6^ M (10.02 kBq mL^−1^) for CEM III/C and [^14^CO_3_^2−^] = 4.5 × 10^−6^ M (10.34 kBq mL^−1^) for CEM III/C + CaCO_3_. For the sake of simplicity, the notation ^14^C has been used throughout the text instead of referring to ^14^CO_3_^2−^.

NaCl EMSURE (≥99.5%), Na_2_SO_4_ EMSURE (≥99.5%), NaOH and KOH Titrisol were obtained from Supelco and Merck, respectively. Ca(OH)_2_ EMSURE was purchased from Merck.

Isosaccharinic acid −1, 4-lactone (ISA_L_) (98.0%) was purchased from Biosynth. Purity of the initial product was verified in a previous study by means of Non-Purgeable Organic Carbon (NPOC), ion chromatography (IC) and ^1^H NMR.^50^

A stock solution of 1 M ISA was prepared by dissolving ISA_L_ in 2 M NaOH, to activate the opening of the ISA carbon chain. Suprapur 65% HNO_3_ (Merck) was used for the dilution of samples for both inductively coupled plasma atomic emission spectroscopy (ICP-OES) and inductively coupled plasma-mass spectrometry (ICP-MS) measurements.

### 2.2 Synthesis and characterization of HCP. Preparation of cement pore water

HCP were prepared using blast furnace slag cement CEM III/C 32.5 N-LH/SR CE with a slag content of 84 wt% provided by ONDRAF/NIRAS.^50^ Two types of cement binders were synthesized in this study with a water to solid ratio equal to 0.46. The first binder was produced by mixing 1440 g of CEM III/C with 660 g of water (referred to as CEM III/C), whereas the second binder was obtained by mixing 95 wt% CEM III/C and 5 wt% of commercial calcite (1440 g) with 660 g of water (referred to as CEM III/C + CaCO_3_). The commercial calcite (Carmeuse, Calcitec 2001M) used in this study is constituted by 99% of carbonates which 98.7% is represented by CaCO_3_. Cement pastes were transferred in 250 mL Kautex bottles, sealed and stored at *T* = (22 ± 2) °C under Ar-atmosphere for a total hydration time of 16 months. In order to provide the sufficient relative humidity during the hydration period, roughly 2 mL of water was added on top of the sample at two days of setting time. After 16 months, both HCPs were crushed and milled by hand to a particle size ≤ 63 µm in an Ar-glovebox. In the latter step, the material selected for the grinding was milled until all the cement powder passed through the 63 µm sieve in order to avoid any size fractionation effect. After hydration, both binders were characterized by X-ray powder diffraction (XRD), thermogravimetric (TGA), and quantitative chemical analysis after alkaline fusion with KOH. XRD measurements were performed with a Bruker D8 Advance X-ray powder diffractometer (Cu Kα radiation, *λ* = 1.54184 Å) in the interval 2*θ* = 2–65°, with incremental steps of 0.012° (2*θ*°) and a total counting time of 2.0 s per step. TGA characterization with a Mettler Toledo TGA/SDTA 8513 was carried out by heating approximately 50 mg of sample within the temperature range 30–1000 °C and with a heating rate of 20 K min^−1^, in a N_2_ atmosphere. XRF (X-ray fluorescence) and quantitative XRD analyses (Rietveld method) of the same non-hydrated CEM III/C were recently reported by Jo and co-workers.^50^

The specific surface area (*A*_s_) of the HCP was determined with gas (N_2_) adsorption experiments following the BET method.^51^ N_2_-BET measurements were performed on 3P instruments micro 200 BET surface area and porosity analyzer. Samples were dried at 60 °C under vacuum for *ca.* 12 hours before measurements. Multi-point BET measurements were performed varying nitrogen *p*/*p*_0_ from 0.05 to 0.30. The specific surface area of CEM III/C and CEM III/C + CaCO_3_ materials investigated in this study are reported in Table 1. For comparison purposes, Table 1 provides also BET data reported for CEM I in.^37^

**Table 1:** Specific surface area (*A*_s_) of CEM III/C, CEM III/C + CaCO_3_, and CEM I as quantified by BET measurements. Errors are determined as two times the standard deviation of three independent measurements

Cement type	*A*_s_ (m^2^ g^−1^)	Reference
CEM III/C	(15.9 ± 2.0)	This study
CEM III/C + CaCO_3_	(17.4 ± 1.6)	This study
CEM I	(12.3 ± 1.6)	37

The pore water composition reported in Jo *et al.* for CEM III/C and CEM III/C + CaCO_3_ using the squeezing method was considered in this work for the recipe of the synthetic pore water compositions:^50^ (i) CEM III/C: 30.3 g of 2 M NaOH, 38.5 g of 2 M KOH, 0.12 g of NaCl, 0.51 g of Na_2_SO_4_, 0.07 g of Ca(OH)_2_; (ii) CEM III/C + CaCO_3_: 29.0 g of 2 M NaOH, 35.7 g of 2 M KOH, 0.21 g of NaCl, 0.45 g of Na_2_SO_4_, 0.06 g of Ca(OH)_2_. Given the low concentrations of Al, Si and carbonate in the squeezed pore water, these components were disregarded in the designed recipe. The synthetic pore waters were equilibrated with the respective hydrated binders by fixing the S/L ratio to 1, 5 and 10 g L^−1^. The final composition of both cement pore waters after equilibration with the corresponding hydrated binders is provided in Table 2, as determined by combination of ICP-OES (PerkinElmer, Optima8300) and IC (ICS-3000, Thermo Scientific™) analyses. Phase separation was achieved by 10 kDa ultrafiltration (Nanosep, Pall Life Sciences).

**Table 2:** Pore water composition of hydrated cement pastes (CEM III/C, CEM III/C + CaCO_3_) as determined in Jo *et al.*^50^ by the squeezing method, and obtained in this work after contacting synthetic pore water with the corresponding binder (see text). All measured values (except pH) reported in molar (M) units

Cement type	Pore water	pH	[Na]	[K]	[Ca]^c^	[SO_4_^2−^]	[Cl^−^]
CEM III/C	Squeezing method^a^	13.0	0.070	0.077	2.9 × 10^−3^	3.62 × 10^−3^	2.1 × 10^−3^
	Synthetic	13.1^d^	(0.073 ± 0.002)	(0.086 ± 0.036)	(1.04 ± 0.03) × 10^−3^	(3.72 ± 0.05) × 10^−3^	(2.20 ± 0.20) × 10^−3^
CEM III/C + CaCO_3_	Squeezing method^b^	13.0	0.068	0.071	2.4 × 10^−3^	3.2 × 10^−3^	3.6 × 10^−3^
	Synthetic	13.0^d^	(0.069 ± 0.005)	(0.075 ± 0.010)	(1.03 ± 0.02) × 10^−3^	(3.20 ± 0.11) × 10^−3^	(3.63 ± 0.10) × 10^−3^

a [Al] = 1 × 10^−4^ M, [Si] = 9 × 10^−5^ M, [CO_3_^2−^] = 6.6 × 10^−4^ M; Al, Si and CO_3_^2−^ were excluded in the preparation of CEM III/C synthetic pore water due to their very low concentrations obtained with the squeezing method. Measured element concentration and pH have a measurement error of ± 5% and ± 0.1 pH units, respectively.

b [Al] = 8 × 10^−5^ M, [Si] = 8 × 10^−5^ M, [CO_3_^2−^] = 9.5 × 10^−4^ M; Al, Si and CO_3_^2−^ were excluded in the preparation of CEM III/C + CaCO_3_ synthetic pore water due to their very low concentrations obtained with the squeezing method. Measured element concentration and pH have a measurement error of ± 5% and ± 0.1 pH units, respectively.

c Decreased Ca concentrations were used in synthetic pore water to avoid oversaturation conditions with respect to portlandite.

d Calculated based on the total concentration of KOH and NaOH added.

### 2.3 Sorption experiments in the absence and presence of ISA and chloride

All sorption experiments were prepared with a total volume of synthetic cement pore water of 10 mL, contacted with the corresponding amount of hydrated cement powder grinded and sieved to a particle size diameter *d* ≤ 63 µm. Suspensions were stored in 15 mL polypropylene vials (Sarstedt) and kept under continuous agitation (100–150 rpm) by means of orbital shaker (VXR basic Vibrax, IKA) to promote mixing between aqueous and solid phases, and to avoid partial or total sedimentation of the solid phase on the vial walls. Cement-^14^C binary system was investigated (i) varying the S/L (1, 5 and 10 g L^−1^) at constant tracer concentration ([^14^C]_tot_ = 8.6 × 10^−9^ M, or 20 Bq mL^−1^), and (ii) under the variation of the initial ^14^C concentration (2.2 × 10^−9^ M ≤ [^14^C]_tot_ ≤ 8.6 × 10^−8^ M) at constant S/L (1 g L^−1^).

The impact of ISA on the uptake of ^14^C by both binders was investigated at constant initial ^14^C concentration ([^14^C]_tot_ = 4.3 × 10^−8^ M, 100 Bq mL^−1^) and S/L (10 g L^−1^), under the variation of ligand concentration (10^−5^ M ≤ [ISA]_tot_ ≤ 0.1 M). In all cases, the addition of the individual components followed the order “(cement + ^14^C) + ISA”, with cement and ^14^C being contacted for two days before the addition of ISA.

The impact of chloride on the retention of ^14^C by both binders was investigated with [Cl^−^]_PW_ ≤ [NaCl]_tot_ ≤ 2 M, where the lower limit corresponds to the chloride concentration measured in the cement pore water (see Table 2). Prior to the addition of the ^14^C tracer, the suspensions containing NaCl were equilibrated for 3 days. Sorption experiments in the presence of NaCl were conducted with [^14^C]_tot_ = 8.6 × 10^−9^ M (20 Bq mL^−1^) and S/L = 1 and 10 g L^−1^. The combined effect of chloride and ISA on the retention of ^14^C was also investigated in a series of sorption experiments with [^14^C]_tot_ = 4.3 × 10^−8^ M (100 Bq mL^−1^), [ISA]_tot_ = 1 × 10^−2^ M, [Cl^−^]_PW_ ≤ [NaCl]_tot_ ≤ 2 M and S/L = 5 g L^−1^. The suspension containing NaCl system was equilibrated for 3 days, followed by the addition of ^14^C and a contact time of 2 days before the final addition of ISA.

In all sorption experiments, phase separation was achieved by ultracentrifugation (90 000 rpm) using an OPTIMA XPN-QC ultracentrifuge and rotor type 90Ti (both Beckman–Coulter). Volumes of *ca.* 4.2 mL were transferred into polypropylene tubes (Beckman–Coulter) and heat-sealed afterwards using a tube topper device (Beckman–Coulter, Tube Topper Kit, 60 Hz). After phase separation, ^14^C activity was quantified by means of liquid scintillation counting (LSC) using a Tri-Carb 3110 TR equipment. LSC samples were prepared with variable sample volumes (0.2–1 mL), mixed with 10 mL of scintillator solution (Hionic-Fluor, PerkinElmer) and 1.5 mL of 2 M NaOH. The latter step is required to avoid the loss of ^14^C through degassing of ^14^CO_2_ due to the acidity of the LSC cocktail. To determine the absolute sample activity (DPM), the measured activity (CPM) was corrected using the appropriate counting efficiency, as reported elsewhere.^49^ The detection limit for ^14^C determined by LSC was ≈ 0.6 Bq mL^−1^ (2.6 × 10^−11^ M) for both CEM III/C and CEM III/C + CaCO_3_.

Experimental conditions of all sorption experiments are summarized in Table 3. Contact times in the table refer to the time after the addition of the radiotracer.

**Table 3:** Summary of the experimental conditions in the binary (cement-^14^C), ternary (cement-^14^C-ISA), (cement-^14^C–NaCl) and quaternary (cement-^14^C-ISA-NaCl) sorption batch experiments performed in the present work

System	S/L (g L^−1^)	Activity of ^14^CO_3_^2−^ (Bq) ([^14^CO_3_^2−^]_tot_ (M))	[NaCl]_tot_ (M)	[ISA]_tot_ (M)	Contact time^a^ (days)	Type of experiment
Cement-^14^C	1, 5, 10	200 (8.6 × 10^−9^)	—	—	2–60	Effect of the S/L
Cement-^14^C	1	50–2000 (2.2 × 10^−9^–8.6 × 10^−8^)	—	—	2–120	Effect of the ^14^C concentration
Cement-^14^C-ISA	10	1000 (4.3 × 10^−8^)	—	10^−5^–0.1	4–120	Effect of [ISA]_tot_
Cement-NaCl–^14^C	1	200 (8.6 × 10^−9^)	[Cl^−^]_PW_^b^–2	—	2–60	Effect of [NaCl]_tot_
Cement-NaCl–^14^C	10	200 (8.6 × 10^−9^)	[Cl^−^]_PW_^b^–2	—	134	Effect of [NaCl]_tot_
Cement-NaCl–^14^C-ISA	5	1000 (4.3 × 10^−8^)	[Cl^−^]_PW_^b^–2	10^−2^	10–120	Combined effect of [NaCl]_tot_ and [ISA]_tot_

a Cement refers to both CEM III/C and CEM III/C + CaCO_3_ binder.

b [Cl^−^]_PW_ refers to the chloride concentration in cement pore solution of CEM III/C (2.20 × 10^−3^ M) and CEM III/C + CaCO_3_ (3.63 × 10^−3^ M) (see Table 2).

The retention of ^14^C by hydrated cement was quantified in terms of distribution ratios (*R*_d_, in L kg^−1^) as a function of equilibration time, S/L, [ISA]_tot_ and [NaCl]_tot_. *R*_d_ values, are calculated as described in eqn (1):1
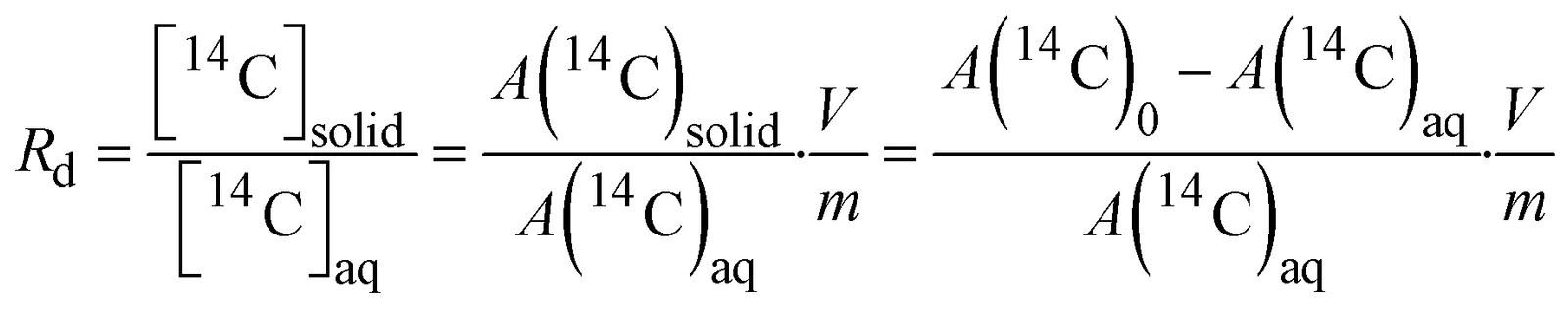
where *A*(^14^C)_0_ is the initial ^14^C activity, *V* is the volume of the solution sample (L) and *m* is the mass of solid phase (kg). *R*_d_ values were quantified by measuring *A*(^14^C)_0_ and *A*(^14^C)_aq_ (activity in the aqueous phase, after phase separation). Uncertainties in *R*_d_ values were calculated as two times the standard deviation of three sample replicates.

In this study, the term *R*_d_ is used instead of *K*_d_ (both in L kg^−1^) because the measured solid-to-solution distribution ratios were determined after a fixed equilibration period and do not necessarily represent true thermodynamic equilibrium.

## 3 Results and discussion

### 3.1 Characterization of hydrated cements

**Fig. 1:**
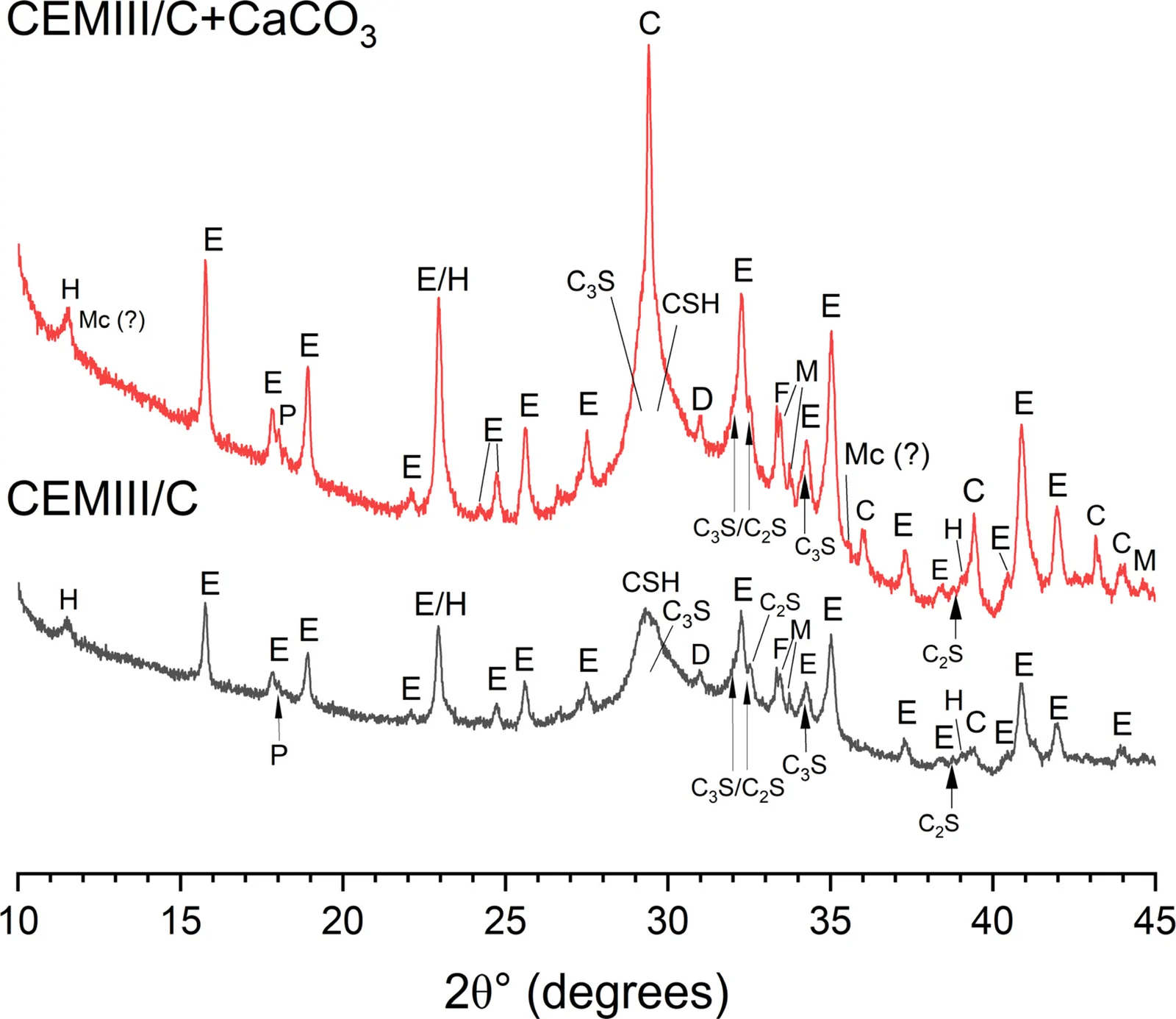
XRD characterization of hydrated CEM III/C (black diffractogram) and CEM III/C + CaCO_3_ (red diffractogram). C: calcite (CaCO_3_, PDF 05-0586), C_2_S (Ca_2_SiO_4_, PDF 31-0299), C_3_S (Ca_3_SiO_5_, PDF 17-0445), D: dolomite (CaMg(CO_3_)_2_, PDF 36-0426), E: ettringite (Ca_6_Al_2_(SO_4_)_3_(OH)_12_·26H_2_O, PDF 41-1451), F: calcium–magnesium–iron–silicate (Ca_2_Fe_(2−2*x*)_Mg_*x*_Si_*x*_O_5_ (*x* = 0.4), PDF 45-0571); H: hydrotalcite (Mg_6_Al_2_CO_3_(OH)_16_·4H_2_O, PDF 14-0191), M: merwinite (Ca_3_Mg(SiO_4_)_2_, PDF 35-0591), Mc: monocarbonate (Ca_4_Al_2_(CO_3_)(OH)_12_·5H_2_O, PDF 41-0219), P. portlandite (Ca(OH)_2_, PDF 44-1481).

XRD diffractograms of both hydrated CEM III/C and CEM III/C + CaCO_3_ are reported in Fig. 1. Despite the relatively minor content of Portland cement (<15 wt%) in the CEM III/C binder used in this work, reflections of unreacted alite (3CaO·SiO_2_ or C_3_S) and belite (2CaO·SiO_2_ or C_2_S) were identified in both binders. Merwinite (Ca_3_Mg(SiO_4_)_2_) has been also identified,^4,16,52^ as well as of calcium–iron–magnesium–silicate (Ca_2_Fe_1.2_Mg_0.4_Si_0.4_O_5_), possibly arising from the solid solution between akerminite (Ca_2_MgSi_2_O_7_)^16^ and Fe_2_O_3_. In addition to less crystalline phases and unreacted slag, both hydrated binders are characterized by the presence of the following crystalline phases: ettringite (Ca_6_Al_2_(SO_4_)_3_(OH)_12_·26H_2_O), hydrotalcite (Mg_6_Al_2_CO_3_(OH)_16_·4H_2_O), dolomite (CaMg(CO_3_)_2_) and calcite (CaCO_3_). As expected, the latter phase is present in larger amounts for the CEM III/C + CaCO_3_ binder, as determined by thermogravimetric analysis (4.6 g of calcite/100 g of sample) compared to CEM III/C (1.0 g of calcite/100 g of sample) reported in the ESM (Electronic Supplementary Material). The broad signal at 2*θ* ≈ 30° belongs to C–S–H phases,^53–55^ possibly defined by a lower Ca/Si ratio and higher Al/Si compared to hydrated OPC, as expected for blended cements with high level of SCM substitution.^17,19,20,22,24,25^ Given the high Al_2_O_3_ content in the unhydrated material,^50^ Al incorporation into the C–S–H phase structure is expected. For both binders, only traces of portlandite can be inferred from the small feature at 2*θ* ≈ 18° (Fig. 1). The very small fraction of portlandite is consistent with the high slag content of CEM III/C-based binders, as portlandite reacts with slag to form C–S–H, leading to a larger inventory of C–S–H phases compared to OPC.^20,22,27^ The presence of AFm phases (*e.g.* monosulfate, monocarbonate, hemicarbonate) is also expected for both binders,^19,56^ but unequivocal phase identification is challenging due to their poor crystallinity and the overlap of XRD and TGA signals. Hence, the small and broad features at 2*θ* ≈ 11.7° are tentatively assigned to hydrotalcite (H) and monocarbonate (Mc). The presence of calcite has been reported to increase the reactivity of slag by decreasing the Al concentration in solution.^31^ This may contribute to the stabilization of monocarbonate (over monosulfonate), ettringite and hydrotalcite compared to the calcite-free system. We note that Lothenbach and co-authors reported also the stabilization of monocarbonate and ettringite over time for OPC blended with 4 wt% limestone.^28^ Furthermore, thermodynamic calculations conducted by Lothenbach and co-workers for the assemblage of OPC with increasing GGBFS content agree well with the phase identification reported in Fig. 1.^17^ These model calculations predict the formation of hydrotalcite-like phases due to the high content of MgO present in the slag, portlandite destabilization at high replacement levels (GGBFS > 70%), disappearance of monocarbonate (GGBFS > 90%) and formation of C–S–H phases with low Ca/Si ratio.

TGA characterization summarized in the ESM supports the presence of ettringite with a weight loss at ≈ 100 °C, which however overlaps with the dehydration of C–S–H and C–A–S–H phases. The presence of hydrotalcite is also hinted by the H_2_O weight loss at ≈ 250 °C and ≈ 400 °C, whereas the weight loss at ≈ 150 °C possibly indicates the presence of AFm phases such as mono- or hemicarbonate.^57^ For temperatures *T* > 600 °C the carbonates weight loss accounts for 0.4 wt% and 1.9 wt% for CEM III/C and CEM III/C + CaCO_3_, respectively.

### 3.2 Uptake of ^14^C by HCP in the absence of ISA and NaCl

**Fig. 2:**
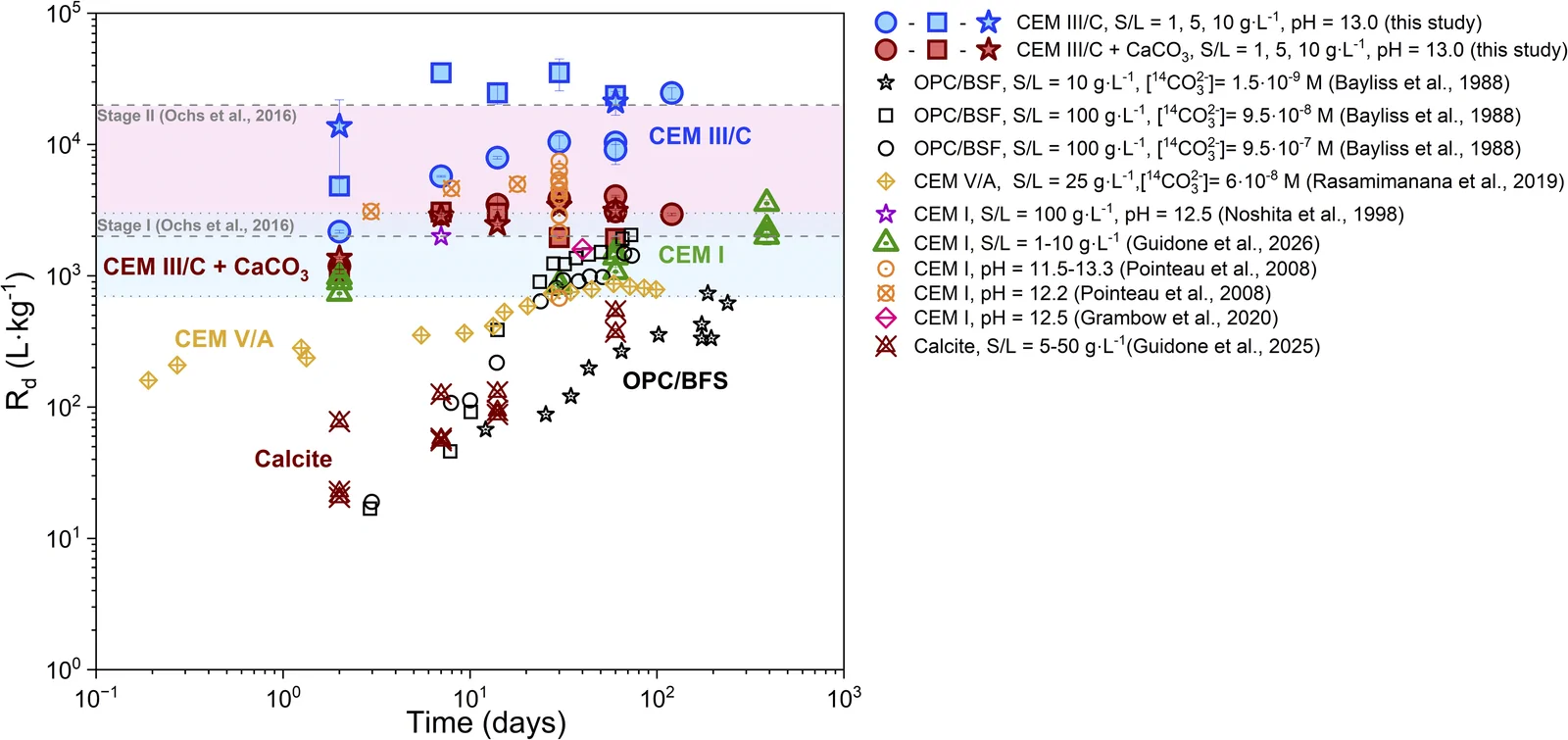
Sorption kinetics for the uptake of ^14^C uptake by CEM III/C (blue) and CEM III/C + CaCO_3_ (red) for three systems with S/L = 1, 5 and 10 g L^−1^ (showed in circle, square and star symbols), obtained in the time frame 2 ≤ *t* (days) ≤ 120. Blue and red regions corresponds to the first and second stages of cement degradation reported by Ochs *et al.*^1^ Black and yellow empty symbols report the ^14^C retention by OPC/BFS and CEM V/A obtained by Bayliss *et al.*^36^ and Rasamimanana *et al.*,^38^ respectively. Empty green and brown symbols show the sorption kinetics of ^14^C by CEM I and calcite carried out by Guidone *et al.*^37,49^ Sorption kinetics of ^14^C by CEM I investigated by Noshita *et al.*,^59^ Pointeau *et al.*^34^ and Grambow *et al.*^60^ are also reported.

Fig. 2 summarizes all sorption kinetics data collected in this work for the uptake of ^14^C by CEM III/C and CEM III/C + CaCO_3_. At short contact times (*t* = 2 days), similar retention values (*R*_d_ ≈ 10^3^ L kg^−1^) were obtained for both CEM III/C (at low S/L ratio) and CEM III/C + CaCO_3_ systems. However, ^14^C retention by CEM III/C shows a more remarkable increase with S/L and time compared to CEM III/C + CaCO_3_, with *R*_d_ ≈ 10^4^ L kg^−1^ after 120 days of contact time. The differences observed between both binders in terms of overall uptake (*R*_d_) and different kinetic trends can be possibly attributed to the different assembly of carbonate-containing phases in both binders, *e.g.*, calcite, hydrotalcite or monocarbonate, as well as to differences in the corresponding recrystallization rates as main driving force for isotopic exchange. Differences in the AFm phase distribution were also evidenced in our recent work dealing with the uptake of ^36^Cl^−^ by CEM III/C and CEM III/C + CaCO_3_.^56,58^ Moreover, although the comparison between both anions (^36^Cl^−^ and ^14^CO_3_^2−^) should be done with care, we note that the retention of ^36^Cl^−^ by CEM III/C + CaCO_3_ (at low NaCl concentrations) was also weaker than for CEM III/C.

As reported for the retention of ^14^C by CEM I,^33,34,37^ the uptake of ^14^C cannot be solely interpreted considering the isotopic exchange with carbonate-containing phase but must account as well for adsorption processes on the positively charged sites of C–S–H phases.^3,32^ Note that hydrated CEM III/C and CEM III/C + CaCO_3_ binders prepared in this work show very similar BET surface areas (15.9 m^2^ g^−1^ and 17.4 m^2^ g^−1^, respectively), and thus differences in the sorption behaviour with time of both binders are unlikely caused by differences in the C–S–H content and surface properties.

Fig. 2 shows also sorption kinetic data reported in the literature for OPC/BFS mixtures,^36^ CEM I,^34,37,59,60^ CEM V/A^38^ and calcite.^49^ In most cases, an increase of *R*_d_ with time can be observed, thus supporting the relevance of isotopic exchange and recrystallization processes as retention mechanisms. For OPC/BFS mixtures, Bayliss and co-workers reported the increase of *R*_d_ values with increasing S/L,^36^ similarly to our observations with the CEM III/C system. Bradbury and Sarott were able to describe the uptake of ^14^C by sulfate resistant Portland cement assuming as uptake mechanism isotopic exchange with finely divided calcite present in the HCP.^39^ Considering the large fraction of calcite in hydrated CEM III/C + CaCO_3_, this binder should expectedly result in greater *R*_d_ values for the uptake of ^14^C than CEM III/C. This is not validated by our experimental results, pointing again towards the involvement of other carbonate-bearing phases, the formation of additional carbonate bearing phases with time and/or other processes in the uptake of ^14^C. We note that *R*_d_ values determined in this work for both CEM III/C and CEM III/C + CaCO_3_ are close to (or slightly above) the upper limit (*R*_d_ = 3000 L kg^−1^) selected in the reference book by Ochs *et al.* for the uptake of ^14^C by cement in the degradation stage I (ref. 1) (Fig. S3, ESM).

**Fig. 3:**
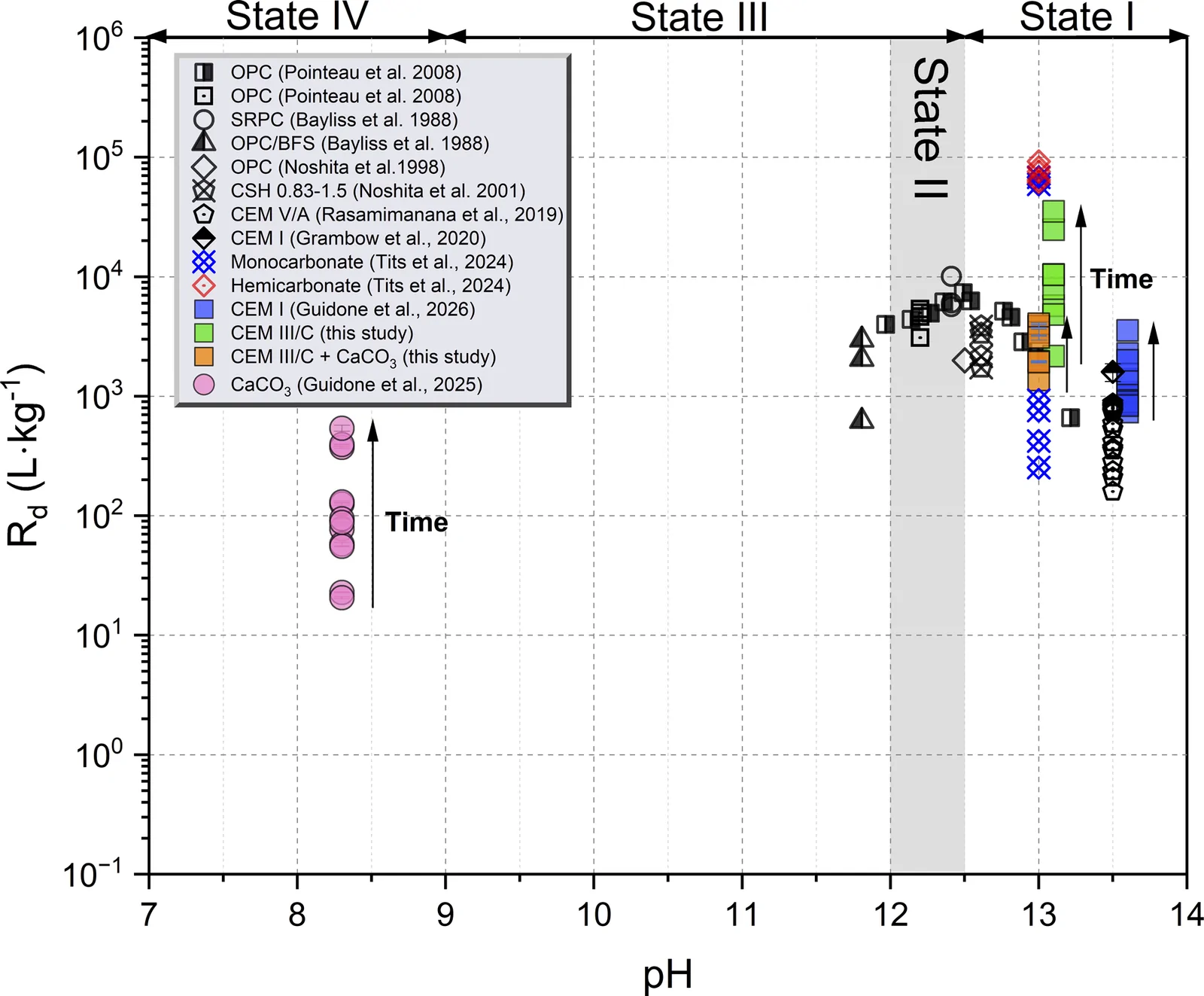
Distribution ratio, *R*_d_, determined for the uptake of inorganic ^14^C by hydrated cement,^34,36–38,59,60^ cement phases (*e.g.* C–S–H phases, AFm phases^32,61^) and calcite^49^ as a function of pH.

Fig. 3 offers a complementary representation of Fig. 2, where the *R*_d_ values obtained for the uptake of ^14^C by cement and cement phases (*e.g.* C–S–H phases, monocarbonate) in this work and in other studies^34,36,59,60^ are summarized as a function of reported pH values. Along with the cement degradation process, the change in the composition and pH of the pore water are expected to influence the surface properties of cement. For example, in study conducted by Pointeau *et al.*,^8,34^ higher retention values were observed alongside the increase of pH corresponding to an increase of zeta potential (*ζ* ≈ +20 mV) in the pH range 11.8–12.5. On the other hand, the negative surface charge (*ζ* ≈ −8 mV) expected for higher pH values (13.2), can result in lower retention values. Alongside the surface properties and pore water composition, time is also a relevant parameter for the determination ^14^C retention. Indeed, long experimental times reflect the role of the recrystallization process on the overall sorption mechanism, and often result in higher ^14^C retention values, as observed for hydrated cement,^37^ carbonate-containing phases (*e.g.* monocarbonate^61^) and calcite.^49^

**Fig. 4:**
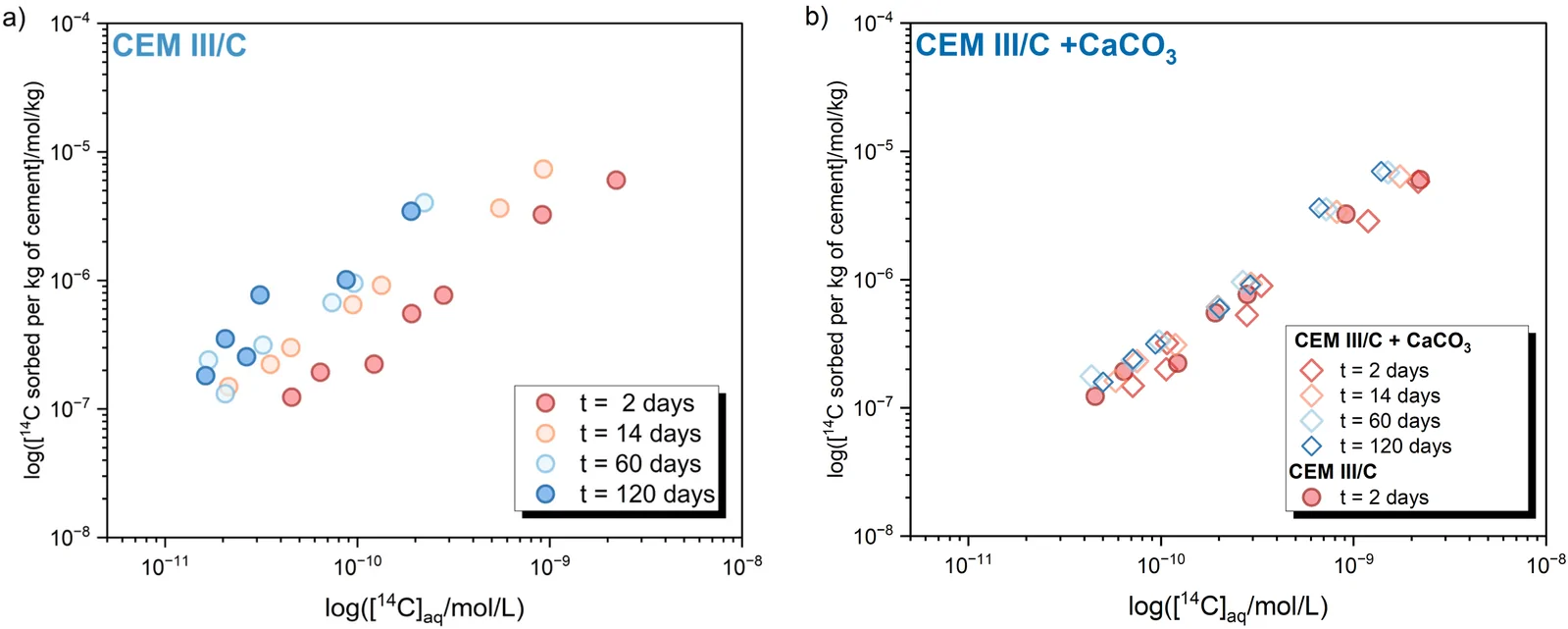
Sorption isotherms for the uptake of ^14^C by HCP, (a) CEM III/C and (b) CEM III/C + CaCO_3_, with a S/L = 1 g L^−1^. Batch sorption experiments were conducted for equilibration times of *t* = 2–120 days.

Fig. 4 shows the sorption isotherms determined for the uptake of ^14^C by CEM III/C and CEM III/C + CaCO_3_. Both systems show a linear sorption behaviour over almost two orders of magnitude in ^14^C concentration. However, as already hinted in the kinetic experiments, sorption isotherms highlight the differential temporal evolution for the uptake of ^14^C by both binders, with a systematic decrease of [^14^C]_aq_ with time for CEM III/C, but an almost time-independent uptake of ^14^C by CEM III/C + CaCO_3_. Fig. 4b confirms the perfect alignment of both sorption isotherms at short contact times (*t* = 2 days), which could be interpreted as a first retention step driven by fast ^14^C adsorption on C–S–H (or C–A–S–H) phases in both binders, followed by slower incorporation/isotopic exchange into carbonate-bearing cement phases (calcite, monocarbonate, hydrotalcite). The different assembly of carbonate phases (and corresponding recrystallization rates) in both binders would then be responsible for the observed differences in the temporal evolution. As carbonate phases are unequivocally present in CEM III/C + CaCO_3_, we hypothesize that they are characterized by slower recrystallization kinetics in the presence of a surplus of calcite, where monocarbonate and carbonate based-containing hydrotalcite had already formed. In contrast, the uptake of carbonates in CEM III/C, where hemicarbonate or hydroxide-containing hydrotalcite is formed, might be faster than the recrystallisation of carbonate-saturated cement phases, in particular as hemicarbonate or hydroxide-containing hydrotalcite are known to form solid solution with carbonate in the interlayer.^62,63^

Thermodynamic modelling was used to calculate the amount of the different hydrates formed. The expected quantities of C–S–H and carbonate-bearing phases in the hydrated cements are compared in Fig. 4, whereas diagrams describing the complete phase distribution of the three hydrated cements are shown in Fig. S3.

**Fig. 5:**
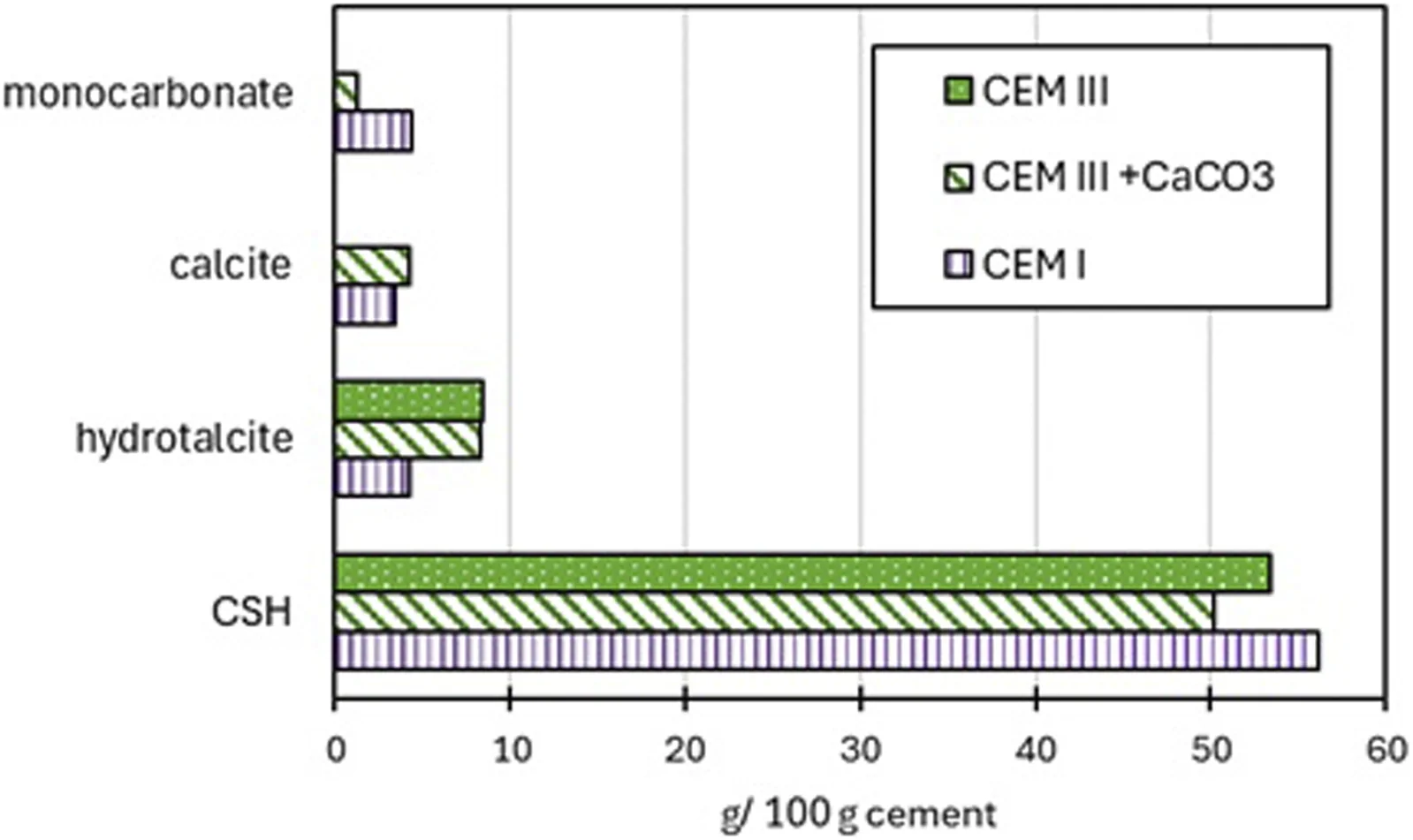
Content of C–S–H phases, hydrotalcite, calcite and monocarbonate in hydrated CEM III/C CEM III/C + CaCO_3_ (composition as detailed in ref. 50) and CEM I (including 4% CaCO_3_, composition from ref. 28). Thermodynamic modelling carried out with GEMS, using the Cemdata18 database,^64^ hydrotalcite phases with carbonate, sulfate or hydroxide from ref. 62 and the CASH + model to describe the uptake of Al and alkalis in C–S–H.^65^ A slag reaction degree of 50% has been assumed.^50^

Fig. 5 shows that the content of C–S–H phases is similar in the three hydrated cements, ranging from ≈ 50 to ≈ 60 g/100 g cement in agreement with the similar BET surface area obtained for the two cements, underlining that the comparable early uptake of carbonates could be mainly related to C–A–S–H. The content of OH- and CO_3_-containing hydrotalcite is similar in CEM III/C and CEM III/C + CaCO_3_, but higher than in CEM I, whereas calcite is only present in CEM III/C + CaCO_3_ and CEM I. Note that XRD suggested the presence of a minor fraction of calcite also in CEM III/C, although this may have caused by carbonation of the original or hydrated material. The presence of minor fractions of monocarbonate is predicted for CEM III/C + CaCO_3_ and CEM I, as qualitatively confirmed by XRD in this work and by Jo and co-workers.^56^

Although experimental observations suggest slower recrystallization rates for the carbonate-bearing phases in hydrated CEM III/C + CaCO_3_, our working hypothesis is that greater *R*_d_ values can be predicted in the long-term for this binder on the basis of its larger inventory of carbonate-bearing phases and their key role in isotopic exchange processes with ^14^CO_3_^2−^. In absolute terms, the initial isotopic ratio ^14^C/C_tot_ in the aqueous phase, the total inventory of carbonate phases in HPC as well as the S/L of a given system will define the upper limit for ^14^C retention.

### 3.3 Uptake of ^14^C in the presence of ISA

**Fig. 6:**
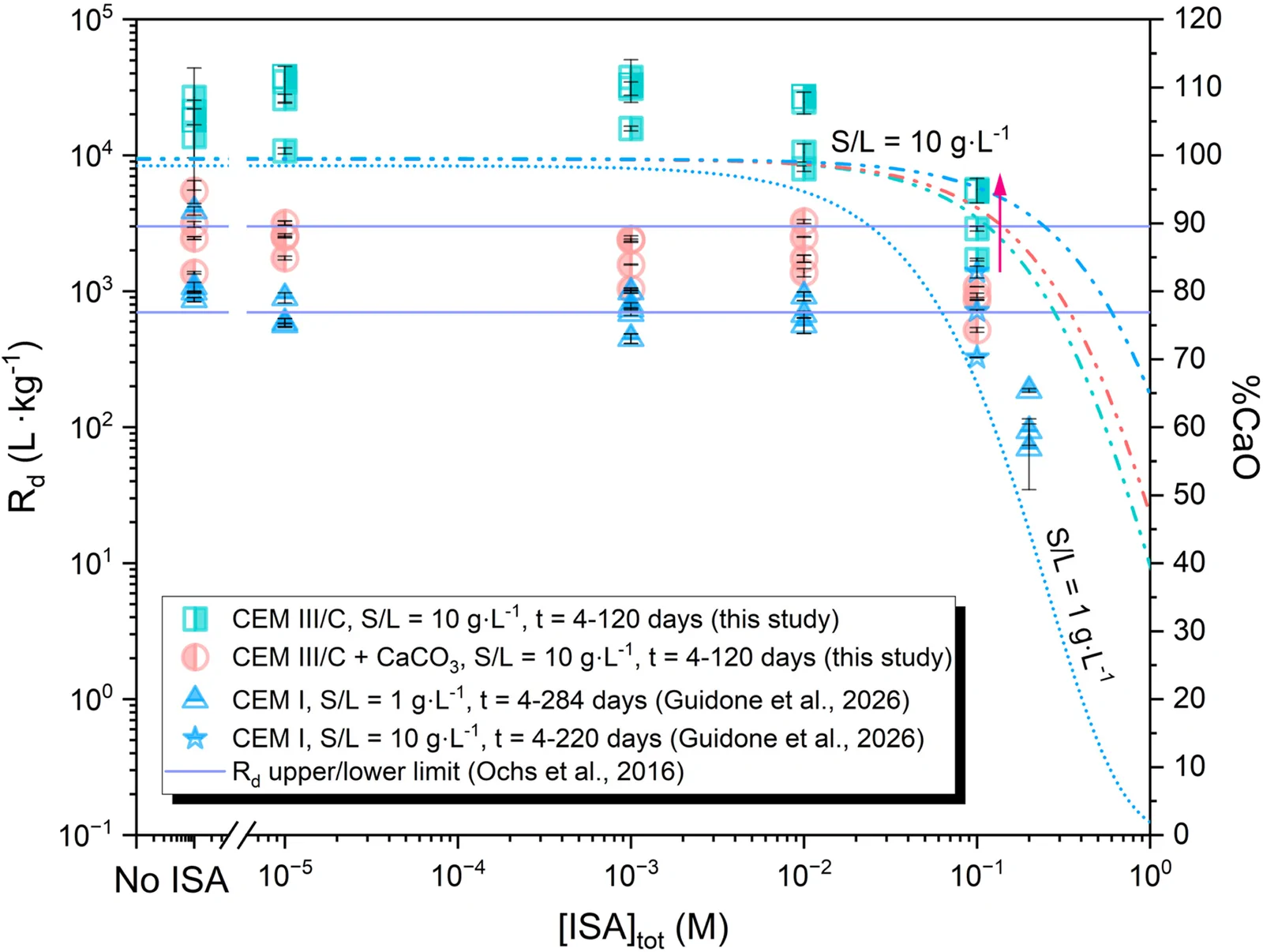
Distribution ratios, *R*_d_, determined for the uptake of ^14^C by HCP in presence of ISA ([ISA]_tot_ = 10^−5^–0.1 M, S/L = 1–10 g L^−1^). Square and circle shaped symbols show the distribution ratios for CEM III/C and CEM III/C + CaCO_3_, respectively. Sorption batch experiments were monitored for an equilibration time of 4 ≤ *t* (days) ≤ 120. Blue symbols report the sorption values by CEM I cement type.^37^ Dotted and double dot-dashed curves correspond to the %CaO in HCP as a function of [ISA]_tot_ obtained for S/L = 1 and 10 g L^−1^, respectively. Solid lines represent the upper and lower *R*_d_ limits reported by Ochs *et al.*^1^ for the degradation stage I of cement (pH = 13.3–12.5).

Fig. 6 shows the impact of ISA on the retention of ^14^C by CEM III/C and CEM III/C + CaCO_3_ as a function of the total ISA concentration added. At ligand concentrations ≤10^−2^ M, distribution ratios determined for the uptake of ^14^C by both binders remain virtually the same as those determined for ISA-free systems. At [ISA]_tot_ > 10^−2^ M, a slight decrease in *R*_d_ is observed for both binders, as compared with ISA-free systems. Similar observations were reported for the retention of ^14^C by CEM I in the presence of ISA,^37^ and are included in Fig. 6 for comparison purposes.

**Fig. 7:**
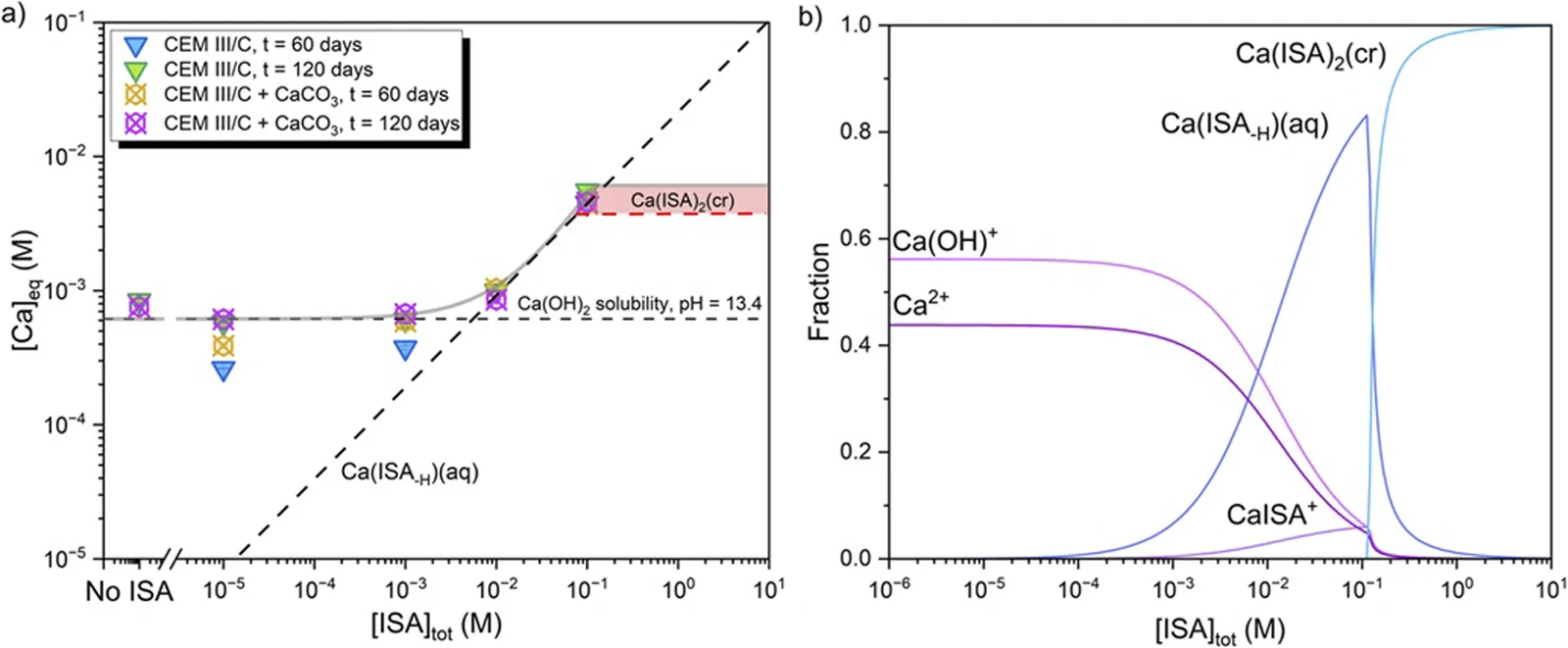
(a) Experimentally measured Ca concentrations in HCP (CEM III/C, full symbols, and CEM III/C + CaCO_3_, empty symbols) systems under increasing ISA concentration (10^−5^ M ≤ [ISA]_tot_ ≤ 0.1 M) at contact times *t* = 60 and 120 days. Solid line corresponds to the solubility of portlandite (Ca(OH)_2_) in equilibrium with increasing amounts of ISA. Dotted red line represents the solubility limit of Ca(ISA)_2_(cr) determined for [Ca] = 5.5 × 10^−3^ M and pH = 13.0. (b) Ca speciation for the solubility curve of Ca(OH)_2_ shown in figure a. Calculations conducted with the ThermoChimie database v.13a.^66^

The presence of ISA leads to the formation of aqueous complexes with Ca, promoting the partial dissolution of cement phases and partially affecting the mineralogy and cement pore water composition (*e.g.* [Ca], [CO_3_^2−^]). Fig. 7a shows the evolution of Ca concentration as a function of [ISA]_tot_, together with solubility calculations considering the formation of Ca-ISA complexes in the aqueous phase. Fig. 7a and b show that the increase of [Ca]_aq_ can be properly explained by the formation of the aqueous complex Ca(ISA_-H_)(aq), where ISA_-H_ represents the ligand with one deprotonated alcohol group. Thermodynamic calculations suggest also the possible precipitation of Ca(ISA)_2_(cr) at [ISA]_tot_ ≥ 0.1 M. Note further that the decrease of [Ca^2+^]_free_ in solution together with the expected sorption of ISA on the cement surface are expected to decrease the surface charge of cement, as demonstrated by Tasi and co-workers for a CEM I system in the degradation stage II.^45^ This process appears to have a relatively minor impact on the retention of ^14^C by CEM III/C and CEM III/C + CaCO_3_.

### 3.4 Uptake of ^14^C in the presence of NaCl and NaCl + ISA

**Fig. 8:**
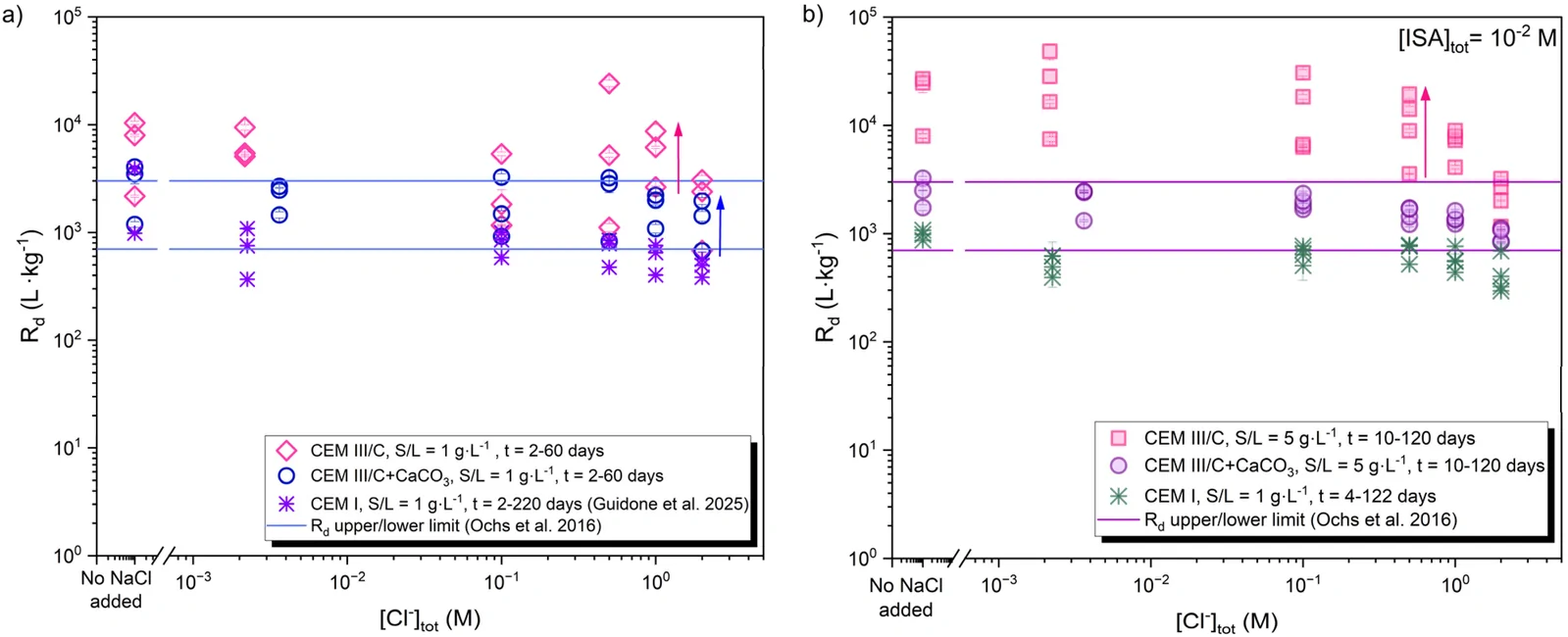
Distribution ratios, *R*_d_, determined for the uptake of ^14^C by HCP (CEM III/C and CEM III/C + CaCO_3_) (a) in the absence and (b) in the presence of ISA ([ISA]_tot_ = 10^−2^ M) as a function of increasing chloride concentration ([NaCl]_tot_ = [Cl^−^]_PW_–2 M) and S/L = 1–10 g L^−1^. ^14^C uptake was monitored for contact times of *t* = 2–134 and 10–120 days in the absence and presence of ISA, respectively. Solid lines represent the upper and lower *R*_d_ limits reported by Ochs *et al.* for the first degradation stage of cement.^1^

The impact of NaCl and the combination of NaCl + ISA on the retention of ^14^C by both binders is reported in Fig. 8a and b, respectively. Both sorption series were carried out by varying the chloride concentration [Cl^−^]_PW_ < [NaCl]_tot_ < 2.0 M, while the ISA concentration used in the cement-NaCl–^14^C-ISA quaternary system was kept constant at [ISA]_tot_ = 10^−2^ M. Less evident impact of NaCl on the retention of ^14^C is observed in ISA-free systems, considering the dispersion of the collected experimental data at different NaCl concentrations (Fig. 8a). In the presence of ISA (Fig. 8b), a minor decrease of the retention of ^14^C could be claimed for both CEM III/C and CEM III/C + CaCO_3_ systems, analogously to the results previously reported for CEM I systems.^37^ NaCl is known to alter both surface properties (*e.g.*, surface charge) and composition of some cement phases,^9,56,67–72^ which in turn may have an impact on the retention of ^14^C. High chloride concentrations lead to the formation of Friedel's salts (Ca_2_(Al,Fe)(OH)_6_Cl·2H_2_O) and consequently to the destabilization of AFm phases,^68,72^ whereas the formation of Kuzel's salt (Ca_4_Al_2_(OH)_12_Cl(SO_4_)_0.5_·5H_2_O) takes also place at lower chloride concentration.^9,73^

**Fig. 9:**
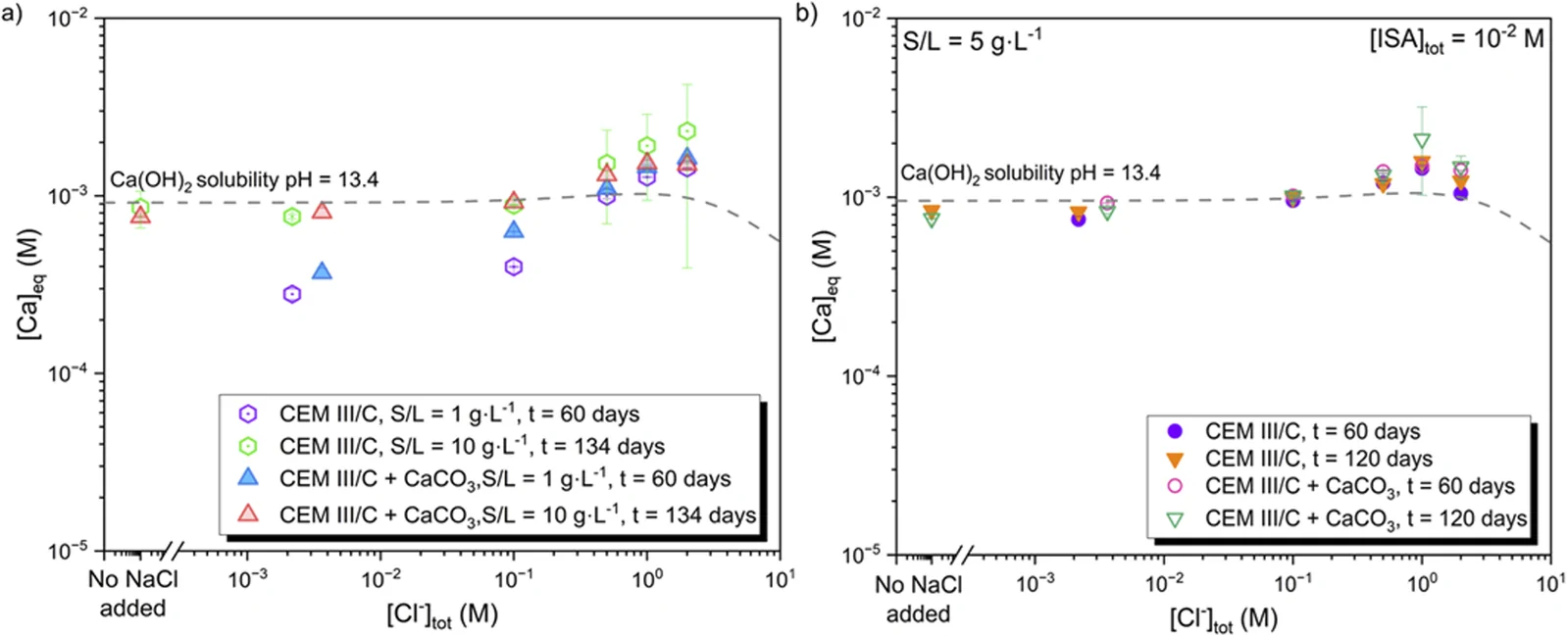
Experimentally measured Ca concentrations in HCP (CEM III/C and CEM III/C + CaCO_3_) systems under increasing NaCl concentrations ([Cl^−^]_PW_ ≤ [NaCl]_tot_ ≤ 2 M), S/L = 1, 5 and 10 g L^−1^ in (a) absence of ISA and (b) in presence of ISA ([ISA]_tot_ = 10^−2^ M) at contact times of *t* = 60, 120 and 134 days. Dashed lines in figures a and b correspond to the solubility of portlandite (Ca(OH)_2_) at pH = 13.4 calculated using the Thermochimie database v.13a and SIT for ionic strength corrections.^66^

The evolution of Ca concentration for both cement-NaCl–^14^C (Fig. 9a) and cement-NaCl–^14^C-ISA (Fig. 9b) is reported in Fig. 9. The weak increase of Ca concentration in solution with increasing NaCl concentration (Fig. 9a) is expectedly linked to the uptake of Na^+^ by C–S–H/C–A–S–H phases.^56,70,74,75^ A decrease in Ca concentrations at high NaCl is predicted as a consequence of ion interaction processes (dashed lines in the figures), although the range of NaCl concentrations considered in this work and the dispersion of the data do not allow to draw definitive conclusions in this respect.

## 4 Conclusions

The retention of inorganic ^14^C by CEM III/C and CEM III/C + CaCO_3_ was investigated by a systematic series of batch sorption experiments at pH ≈ 13. The effect of ISA and chloride on the retention behaviour of ^14^C was further studied on the basis of sorption experiments with ternary (cement-NaCl–^14^C/cement-^14^C-ISA) and quaternary (cement-NaCl–^14^C-ISA) systems.

Solid phase characterization by means of XRD and TGA confirms the presence of some unreacted clinker components (alite, belite), C–S–H phases (expectedly with Al incorporation, *i.e.*, C–A–S–H), ettringite, hydrotalcite, dolomite and calcite. XRD and TGA hint towards the presence of monocarbonate in the hydrated CEM III/C + CaCO_3_ cement, although neither unequivocal identification nor quantification can be granted due to their low content and poorly crystalline character.

The moderate retention of ^14^C observed for both materials at short contact times (*t* = 2 days) is primarily attributed to adsorption on C–S–H (or C–A–S–H) phases, in line with previous studies conducted with different cement materials and cement phases (*e.g.*, CEM I, CEM V, C–S–H phases, *etc.*). A steady increase of the uptake with time is only observed for CEM III/C (where the initial uptake is dominated by C–S–H) but in the presence of additional carbonate, an uptake in the interlayer of hemicarbonate and hydrotalcite might occur relatively rapid. In contrast, (mostly) time-independent *R*_d_ values are determined for CEM III/C + CaCO_3_ within the timeframe of this study (*t* ≤ 120 days). This behaviour is attributed to the presence of calcite, monocarbonate and carbonate containing hydrotalcite in hydrated CEM III/C + CaCO_3_ and different recrystallization rates in the two binders, which accordingly result in different isotopic exchange rates. In the long-term, we hypothesize that *R*_d_ values for the CEM III/C + CaCO_3_ should increase even above those determined for CEM III/C, accounting for the larger inventory of carbonate-bearing phases in the former binder.

The presence of ISA at ligand concentrations ≤ 10^−2^ M has a negligible impact on the uptake of ^14^C by both binders. The minor decrease in *R*_d_ values observed in both cases at [ISA]_tot_ > 10^−2^ M is explained by the slight dissolution of cement phases triggered by the formation of CaISA_-H_(aq) and CaISA_2_(cr), as experimentally measured in terms of Ca concentrations in the aqueous phase, and also predicted by thermodynamic calculations. These observations are in line with previous studies investigating the retention of ^14^C by CEM I and calcite in the presence of ISA.^37,49^ High NaCl concentrations trigger the formation of Friedel's salt and impact surface charge of C–S–H phases, but have a negligible effect on the retention of ^14^CO_3_^2−^ by CEM III/C or CEM III/C + CaCO_3_. The combined effect of ISA and chloride was investigated on the basis of systematic experiments with the quaternary system cement-NaCl–^14^C-ISA. The slight effect on ^14^C uptake observed for this system is attributed to a combination of Ca-ISA complexation and ion–ion interaction processes.

This work provides a quantitative description and improved process understanding for the uptake of ^14^C by CEM III/C and CEM III/C + CaCO_3_ binders, including the effect of ISA and chloride as components expected in certain L/ILW-SL waste streams.

## Author contributions

Rosa Ester Guidone: conceptualization, investigation, methodology, validation, visualization, writing – original draft, writing – review & editing; Nils Huber: investigation, methodology, validation, writing – review & editing; Barbara Lothenbach: validation, visualization, writing – review & editing; Florian Bocchese: supervision, conceptualization, writing – review & editing; Stéphane Brassinnes: supervision, conceptualization, writing – review & editing; Marcus Altmaier: supervision, conceptualization, project administrator, writing – review & editing; Xavier Gaona: supervision, resources, conceptualization, funding acquisition, methodology, project administrator, writing – review & editing.

## Conflicts of interest

There are no conflicts to declare.

## Supplementary Material

RA-016-D6RA04948F-s001

## Data Availability

The data supporting this article have been included as part of the supplementary information (SI). Supplementary information is available. See DOI: https://doi.org/10.1039/d6ra04948f.

## References

[cit1] Ochs M. , Mallants D. and Wang L., Radionuclide and Metal Sorption on Cement and Concrete, Springer, 2016.

[cit2] Yim M.-S., Caron F. (2006). Prog. Nucl. Energy.

[cit3] Wieland E. , Radionuclide Retention in the Cementitious Near-Field of a Repository for Low- and Intermediate-Level Waste: Development of the Cement Sorption Database, National Cooperative for the Disposal of Radioactive Waste Nagra, NTB 23-07, 2023

[cit4] Taylor H. F. , Cement Chemistry, Thomas Telford London, 1997.

[cit5] Tits J. , Fujita T., Harfouche M., Dähn R., Tsukamoto M. and Wieland E., Radionuclide uptake by calcium silicate hydrates: Case studies with Th (IV) and U (VI), Paul Scherrer Institute, PSI Report Nr. 14-03, 2014

[cit6] Viallis-Terrisse H., Nonat A., Petit J.-C. (2001). J. Colloid Interface Sci..

[cit7] Labbez C., Pochard I., Jönsson B., Nonat A. (2011). Cem. Concr. Res..

[cit8] Pointeau I., Reiller P., Macé N., Landesman C., Coreau N. (2006). J. Colloid Interface Sci..

[cit9] Balonis M., Lothenbach B., Le Saout G., Glasser F. P. (2010). Cem. Concr. Res..

[cit10] Ke X., Bernal S. A., Provis J. L. (2017). Cem. Concr. Res..

[cit11] Nedyalkova L., Lothenbach B., Geng G., Mäder U., Tits J. (2020). Appl. Geochem..

[cit12] Lothenbach B., Nedyalkova L., Rojo H., Wieland E., Mäder U., Tits J. (2024). Appl. Geochem..

[cit13] Ma B., Fernandez-Martinez A., Grangeon S., Tournassat C., Findling N., Claret F., Koishi A., Marty N. C., Tisserand D., Bureau S. (2017). Environ. Sci. Technol..

[cit14] Schneider M., Romer M., Tschudin M., Bolio H. (2011). Cem. Concr. Res..

[cit15] CEN-CENELEC , EN 197–1:2000, Cement – Part 1: Composition, Specifications and Conformity Criteria for Common Cements, European Commitee for Standardization, Brussels, Belgium, 2011.

[cit16] Siddique R., Bennacer R. (2012). Resour., Conserv. Recycl..

[cit17] Lothenbach B., Scrivener K., Hooton R. D. (2011). Cem. Concr. Res..

[cit18] Osmanovic Z., Haračić N., Zelić J. (2018). Cem. Concr. Compos..

[cit19] Saillio M., Baroghel-Bouny V., Bertin M., Pradelle S., Vincent J. (2019). Constr. Build. Mater..

[cit20] Escalante-Garcia J. I., Sharp J. H. (2004). Cem. Concr. Compos..

[cit21] Luke K., Lachowski E. (2008). J. Am. Ceram. Soc..

[cit22] Taylor R., Richardson I., Brydson R. (2010). Cem. Concr. Res..

[cit23] Glasser F. , Tyrer M. and Quillin K., The Chemistry of Blended Cements and Backfills Intended for Use in Radioactive Waste Disposal, Environment Agency, Bristol (United Kingdom), 1999.

[cit24] Richardson I., Groves G. (1997). J. Mater. Sci..

[cit25] Richardson I. G. (1999). Cem. Concr. Res..

[cit26] Hemstad P., Lothenbach B., De Weerdt K. (2024). Cem. Concr. Res..

[cit27] Lothenbach B., Le Saout G., Ben Haha M., Figi R., Wieland E. (2012). Cem. Concr. Res..

[cit28] Lothenbach B., Le Saout G., Gallucci E., Scrivener K. (2008). Cem. Concr. Res..

[cit29] Matschei T., Lothenbach B., Glasser F. (2007). Cem. Concr. Res..

[cit30] Damidot D., Stronach S., Kindness A., Atkins M., Glasser F. P. (1994). Cem. Concr. Res..

[cit31] Adu-Amankwah S., Zajac M., Stabler C., Lothenbach B., Black L. (2017). Cem. Concr. Res..

[cit32] Noshita K., Nishi T., Yoshida T., Fujihara H., Saito N., Tanaka S. (2000). MRS Online Proc. Libr..

[cit33] Noshita K., Nishi T., Matsuda M., Izumida T. (1995). MRS Online Proc. Libr..

[cit34] Pointeau I., Coreau N., Reiller P. E. (2008). Radiochim. Acta.

[cit35] Allard B. , Torstenfelt B. and Andersson K., Sorption Behaviour of 14 C in Groundwater/rock and in Groundwater/concrete Environments, Programraadet foer Radioaktivt Avfall, 1981

[cit36] Bayliss S., Ewart F. T., Howse R. M., Smith-Briggs J. L., Thomason H. P., Willmott H. A. (1987). MRS Online Proc. Libr..

[cit37] Guidone R. E., Huber N., Lothenbach B., Bocchese F., Brassinnes S., Altmaier M., Gaona X. (2026). Appl. Geochem..

[cit38] Rasamimanana S. , Landesman C., Ribet S., Perrigaud K., Bessaguet N. and Grambow B., Uptake and diffusion properties of HTO and inorganic C-14 through hardened cement paste: Influence of water saturation and carbonation, in Proceedings of the Third Workshop of the HORIZON 2020 CEBAMA Project, n°D4.13 European Commission Euratom Research and Training Programme, Report Deliverable 2019, pp. 219–230

[cit39] Bradbury M. H. and Sarott F.-A., Sorption Databases for the Cementitious Near-Field of a L/ILW Repository for Performance Assessment, Paul Scherrer Institut, PSI Report Nr. 95-06, 1995

[cit40] Van Loon L., Glaus M., Stallone S., Laube A. (1997). MRS Online Proc. Libr..

[cit41] Glaus M., Van Loon L., Achatz S., Chodura A., Fischer K. (1999). Anal. Chim. Acta.

[cit42] Glaus M. A. and Van Loon L. R., Cellulose degradation at alkaline conditions: Long-term experiments at elevated temperatures, Paul Scherrer Institut, PSI Report Nr. 04-01, 2004

[cit43] Tasi A., Gaona X., Fellhauer D., Böttle M., Rothe J., Dardenne K., Polly R., Grivé M., Colàs E., Bruno J. (2018). Appl. Geochem..

[cit44] Tasi A., Gaona X., Fellhauer D., Böttle M., Rothe J., Dardenne K., Polly R., Grivé M., Colàs E., Bruno J. (2018). Appl. Geochem..

[cit45] Tasi A., Gaona X., Rabung T., Fellhauer D., Rothe J., Dardenne K., Lützenkirchen J., Grivé M., Colàs E., Bruno J. (2021). Appl. Geochem..

[cit46] Kobayashi T., Teshima T., Sasaki T., Kitamura A. (2017). J. Nucl. Sci. Technol..

[cit47] Vercammen K., Glaus M., Van Loon L. R. (2001). Radiochim. Acta.

[cit48] Tits J., Wieland E., Bradbury M. (2005). Appl. Geochem..

[cit49] Guidone R. E., Huber N., Heberling F., Sittel T., Palina N., Bocchese F., Brassinnes S., Altmaier M., Gaona X. (2025). RSC Adv..

[cit50] Jo Y., Çevirim-Papaioannou N., Franke K., Fuss M., Pedersen M., Lothenbach B., de Blochouse B., Altmaier M., Gaona X. (2023). Cem. Concr. Res..

[cit51] Brunauer S., Emmett P. H., Teller E. (1938). J. Am. Chem. Soc..

[cit52] Lothenbach B., Winnefeld F. (2006). Cem. Concr. Res..

[cit53] Guidone R. E., Gaona X., Winnefeld F., Altmaier M., Geckeis H., Lothenbach B. (2024). Cem. Concr. Res..

[cit54] Yan Y., Yang S.-Y., Miron G. D., Collings I. E., L'Hôpital E., Skibsted J., Winnefeld F., Scrivener K., Lothenbach B. (2022). Cem. Concr. Res..

[cit55] L’Hôpital E., Lothenbach B., Le Saout G., Kulik D., Scrivener K. (2015). Cem. Concr. Res..

[cit56] Jo Y., Lothenbach B., Çevirim-Papaioannou N., de Blochouse B., Altmaier M., Gaona X. (2024). Cem. Concr. Res..

[cit57] Lothenbach B. , Durdzinski P. and De Weerdt K., A Practical Guide to Microstructural Analysis of Cementitious Materials, 2016, vol. 1, pp. 177–211

[cit58] Jo Y., Szabo P. G., Guidone R. E., Lothenbach B., Çevirim-Papaioannou N., Coppens E., Altmaier M., Gaona X. (2026). Cem. Concr. Res..

[cit59] Noshita K., Nishi T., Matsuda M. (1998). Waste Manage..

[cit60] Grambow B., López-García M., Olmeda J., Grivé M., Marty N. C., Grangeon S., Claret F., Lange S., Deissmann G., Klinkenberg M. (2020). Appl. Geochem..

[cit61] Tits J., Curti E., Laube A., Wieland E., Provis J. (2024). Appl. Geochem..

[cit62] Bernard E., Zucha W. J., Lothenbach B., Mäder U. (2022). Cem. Concr. Res..

[cit63] Georget F., Lothenbach B., Wilson W., Zunino F., Scrivener K. L. (2022). Cem. Concr. Res..

[cit64] Lothenbach B., Kulik D. A., Matschei T., Balonis M., Baquerizo L., Dilnesa B., Miron G. D., Myers R. J. (2019). Cem. Concr. Res..

[cit65] Miron D. G. , Kulik D. A. and Lothenbach B., Incremental parameterisation of cash+ sublattice solid solution model against recent data for Na, K, and Al uptake in C-S-H, ERICA CASH II final conference, Technische Universität Wien, 2021, pp. 53–54.

[cit66] Madé B., Bower W., Brassinnes S., Colàs E., Duro L., Blanc P., Lassin A., Harvey L., Begg J. (2025). Appl. Geochem..

[cit67] Glasser F. P., Marchand J., Samson E. (2008). Cem. Concr. Res..

[cit68] Jo Y., Androniuk I., Çevirim-Papaioannou N., de Blochouse B., Altmaier M., Gaona X. (2022). Cem. Concr. Res..

[cit69] Jain A., Gencturk B., Pirbazari M., Dawood M., Belarbi A., Sohail M. G., Kahraman R. (2021). Cem. Concr. Res..

[cit70] Hill J., Harris A. W., Manning M., Chambers A., Swanton S. W. (2006). Waste Manage..

[cit71] Luping T., Nilsson L.-O. (1993). Cem. Concr. Res..

[cit72] Nielsen E. P., Herfort D., Geiker M. R. (2005). Cem. Concr. Res..

[cit73] Cao Y., Guo L., Chen B., Wu J. (2020). Constr. Build. Mater..

[cit74] Hong S.-Y., Glasser F. P. (2002). Cem. Concr. Res..

[cit75] Yan Y., Ma B., Miron G. D., Kulik D., Scrivener K., Lothenbach B. (2022). Cem. Concr. Res..

