# Supplementary material for: Uptake of 14CO32− by HCP (CEM III/C and CEM III/C + CaCO3) in the presence and absence of ISA and chloride
Source: RSC Adv. 2026 Aug 21;16(41):49318–30. doi: 10.1039/d6ra04948f (PMC13498421; doi:10.1039/d6ra04948f)
Supplement: Supplementary file 1 [file RA-016-D6RA04948F-s001.pdf]

Supplementary Information (SI) for RSC Advances.
This journal is © The Royal Society of Chemistry 2026

Electronic Supplementary material (ESM)

Uptake of $^{14}CO_3^{2-}$ by HCP (CEM III/C and CEM III/C + $CaCO_3$) in the presence and absence of ISA and chloride

Rosa Ester Guidone[1,*], Nils Huber[1], Barbara Lothenbach[2], Florian Bocchese[3], Stéphane Brassinnes[3], Marcus Altmaier[1], Xavier Gaona[1,*]

[1]*Karlsruhe Institute of Technology (KIT), Institute for Nuclear Waste Disposal (INE), Hermann-von-Helmholtz-Platz 1, 76344 Eggenstein-Leopoldshafen, Germany*

[2]*Laboratory Concrete & Asphalt, Empa, Swiss Federal Laboratories for Materials Science and Technology, Dübendorf, Switzerland*

[3]*ONDRAF/NIRAS, Belgian Agency for Radioactive Waste and Enriched Fissile Materials, Brussels, Belgium*

1. Cement characterization

1.1. TGA analysis

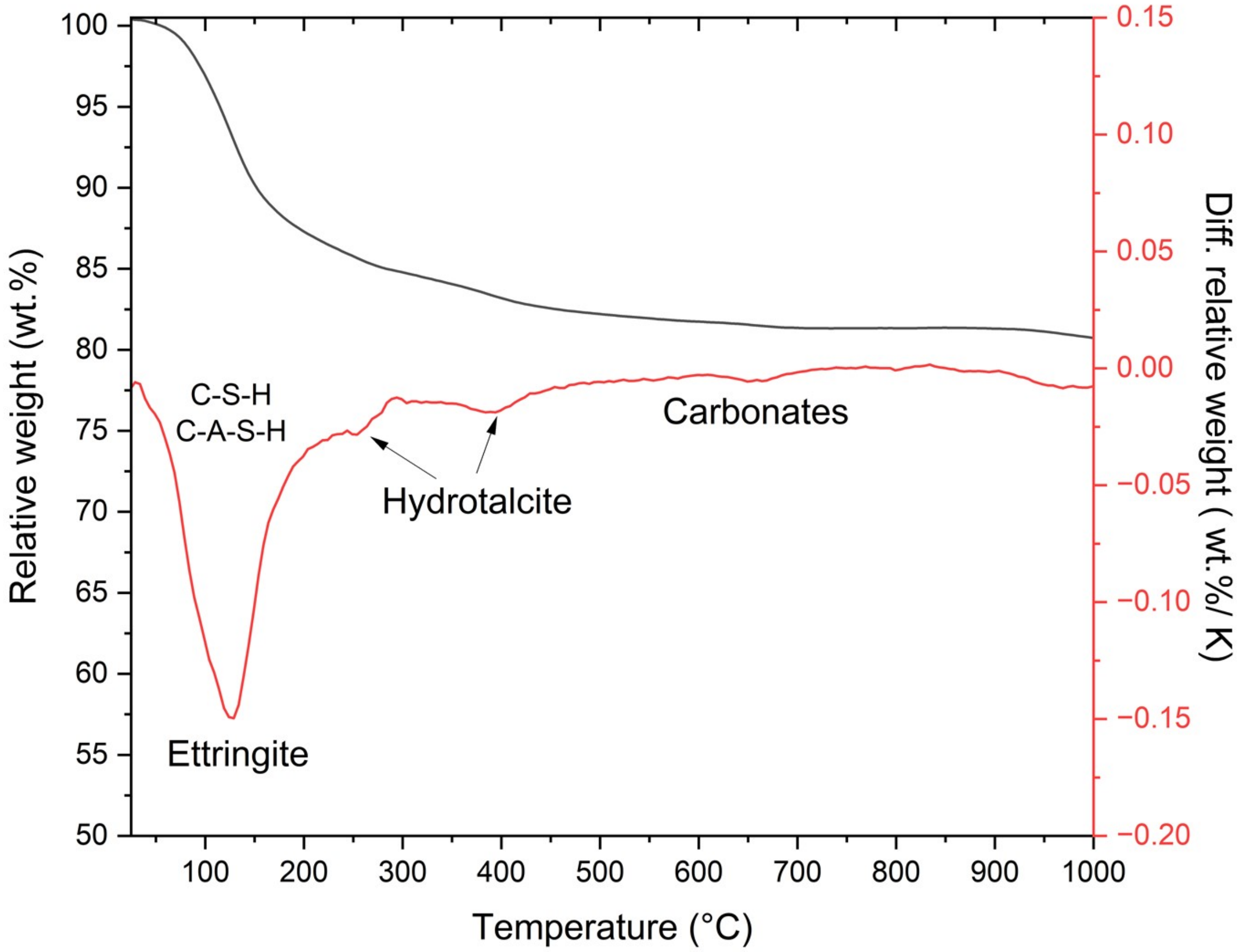

Figure S1. TGA analysis of CEM III/C at 16 months of hydration time.

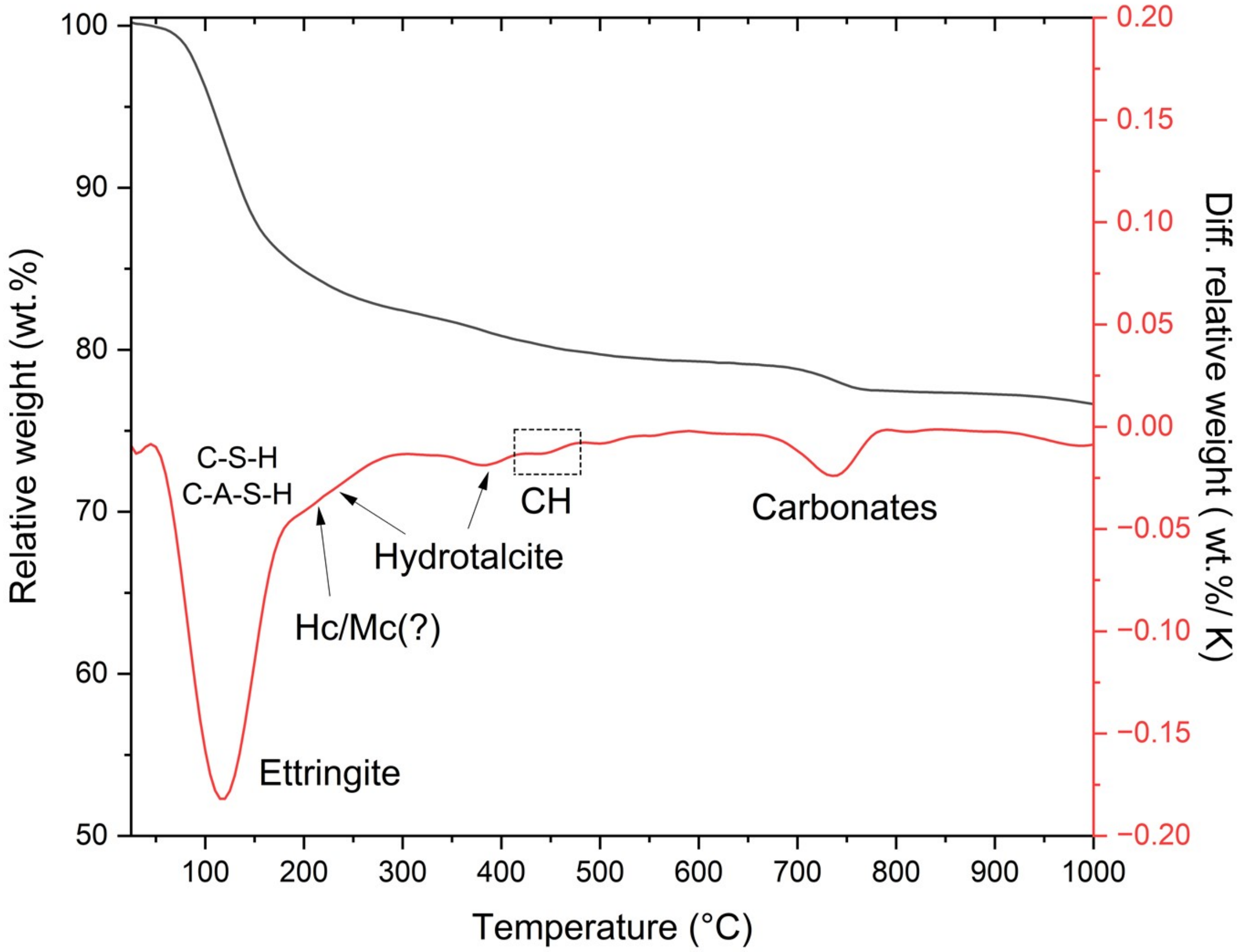

Figure S2. TGA analysis of CEM III/C +$CaCO_3$ at 16 months of hydration time.

1.2. Calcite quantification

Calcite quantification has been carried out according to the approach reported in [1]. Calcite decomposition to CaO and CO_2 occurs at temperatures above 600 °C. The weight loss (WL_{CaCO_3}) caused by de-carbonation process can be used to determine the amount of calcite present in the system knowing the molecular masses of $CaCO_3$ (M_{CaCO_3}) and CO_2 (M_{CO_2}).

$$CaCO_{3,\ measured} = WL_{CaCO_3} \times \frac{M_{CaCO_3}}{M_{CO_2}} \qquad \textit{Eq. 1}$$

To convert the amount of $CaCO_3$ in the measured sample (Eq.1) to calcite per 100 g of cement paste, the following equation (Eq.2) has been used:

$$CaCO_{3,\ paste} = \frac{CaCO_{3,\ measured}}{(1 - H_2O_{bond})(1 + {}^{w}/_{c})} \qquad \textit{Eq. 2}$$

Where *(1-H_2O_{bond})* corresponds to the sample weight at 600°C and *w/c* is the water to cement ratio, which corresponds to 0.46 in this study. Further details are reported in Figure S1.

Table S1. Measured calcite weight loss (WL_{CaCO3}) and water-free sample weight for both CEM III/C and CEM III/C+$CaCO_3$.

	WL_{CaCO3} (wt.%)	Sample weight at 600°C (mg)
CEM III/C[a]	0.44	41
CEM III/C +$CaCO_3$[b]	2.02	49

[a] Initial sample weight:50.8 mg.
[b] Initial sample weight: 49.8 mg.

1.3. Phase composition analysis (GEMS)

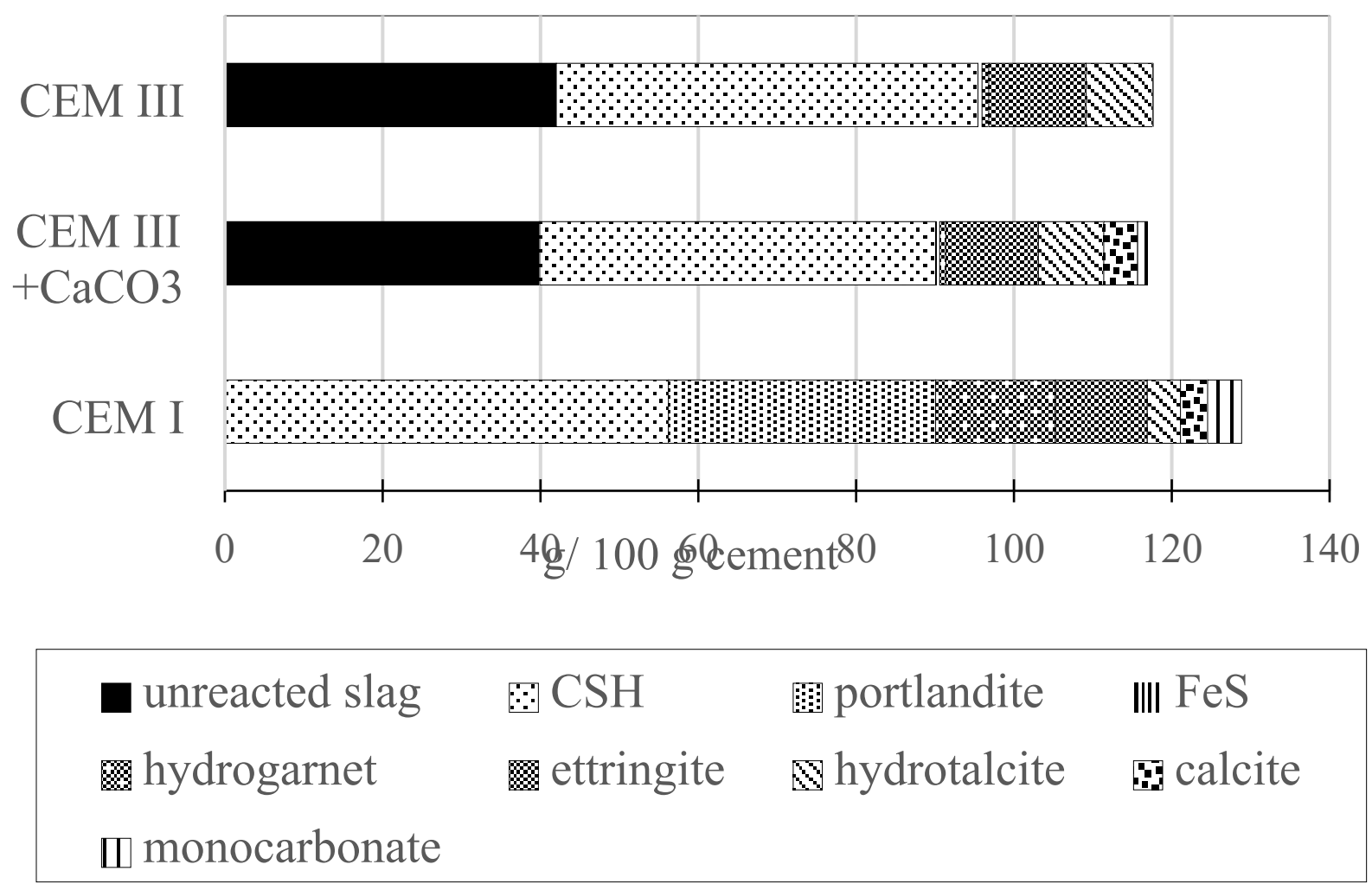

Figure S3. Calculated phase composition of hydrated CEM III/C CEM III/C + $CaCO_3$ (composition as detailed in [2] and CEM I (composition from [3]). Thermodynamic modelling carried out with GEMS, using the Cemdata18 database[4], hydrotalcite phases with carbonate, sulfate or hydroxide from [5], and the CASH+ model to describe the uptake of Al and alkalis in C-S-H[6]. A slag reaction degree of 50% has been assumed.[2]

2. Sorption experiments

2.1. Effect of the S/L ratio

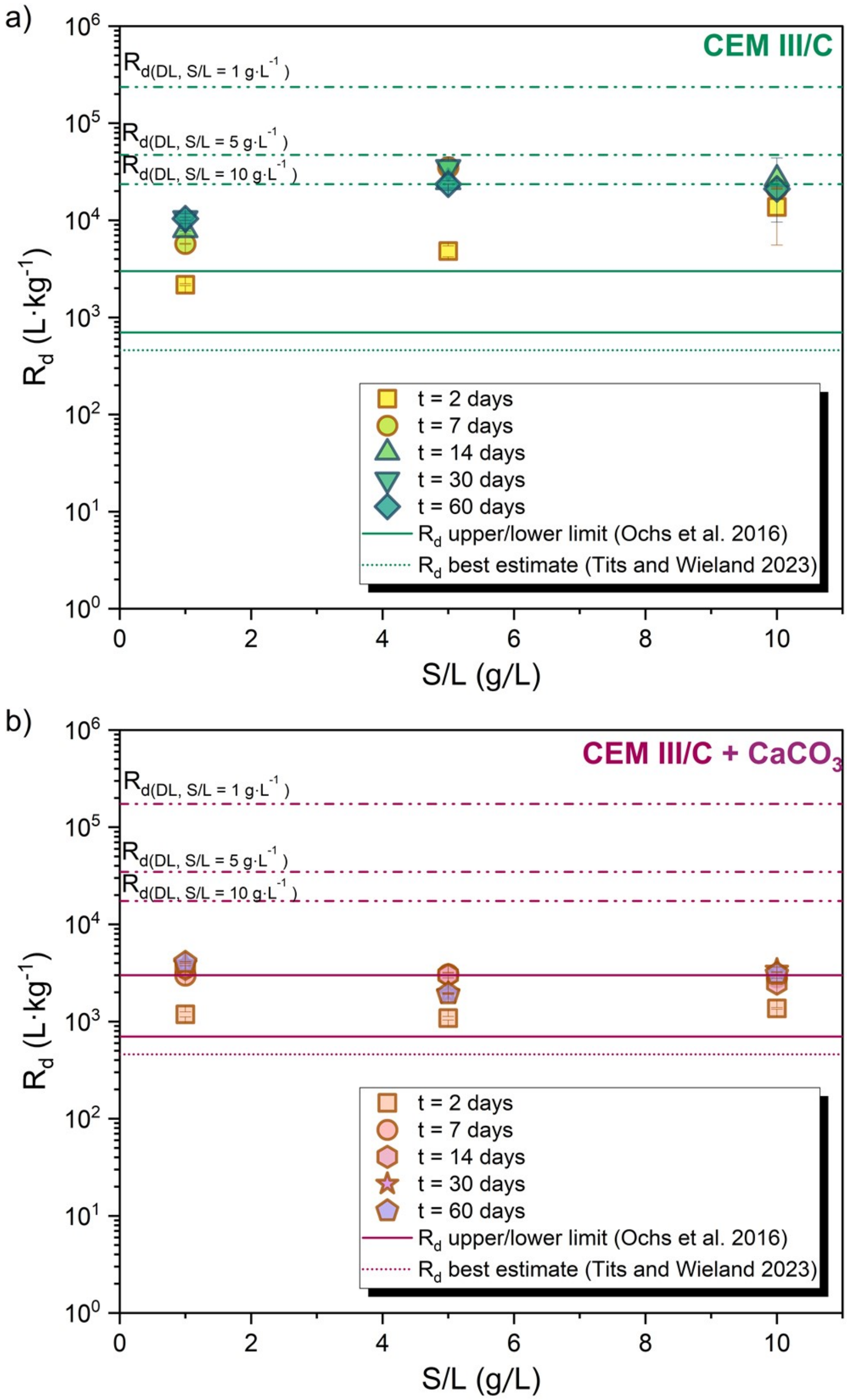

Figure S4. Distribution ratios, R_d, determined in this work for the uptake of ^{14}C by (a) CEM III/C and (b) CEM III/C+$CaCO_3$ as a function of S/L. Solid lines represent the upper and lower R_d limits reported by Ochs et al.[7] for the degradation stage I of cement. Dotted line shows the best estimate for ^{14}C by HCP (cement degradation stage I) reported by [8].Dotted-dashed lines represent the R_d determined at the detection limit (DL) for each system.

2.2. Distribution ratios determined in the sorption experiments

2.2.1. ^{14}C-cement

Table S2. Distribution ratios, R_d, determined in the sorption experiments for the ^{14}C-CEM III/C binary system.

Initial activity, (Bq·mL^{-1})	S/L, (g·L^{-1})	Time, (days)	R_d, (L kg^{-1})	Error[a]	Experiment purpose
20	1	2	2170	34	Sorption kinetics (variation of S/L ratio)
20	1	7	5725	253	
20	1	14	7952	122	
20	1	30	10461	807	
20	1	60	10384	368	
20	5	2	4827	417	
20	5	7	[b]	-	
20	5	14	24775	3002	
20	5	30	35261	6418	
20	5	60	22629	1956	
20	10	2	13737	5461	
20	10	7	[b]	-	
20	10	14	(26737)[c]	(11433)	
20	10	30	[b]	-	
20	10	60	21080	4333	
5	1	2	2714	410	Sorption isotherm (variation of ^{14}C initial activity)
7.5	1	2	3014	216	
10	1	2	4412	126	
20	1	2	2883	20	
30	1	2	2739	62	
100	1	2	3558	443	
200	1	2	2724	16	
5	1	14	6966	1708	
7.5	1	14	6312	688	
10	1	14	6671	353	
20	1	14	6854	322	
30	1	14	6862	79	
100	1	14	6639	36	
200	1	14	7926	45	
5	1	60	6409	1109	
7.5	1	60	14293	19	
10	1	60	9734	1809	
20	1	60	9127	2074	
30	1	60	9960	931	

100	1	60	18100	767	
200	1	60	2950	29	
5	1	120	11149	2942	
7.5	1	120	9570	475	
10	1	120	17061	996	
20	1	120	24679	2449	
30	1	120	11551	660	
100	1	120	18154	291	
200	1	120	6088	185	

[a] Uncertainties are reported as two times the standard deviation (2σ) of mean values.
[b] Measured value below the detection limit.
[c] Measured value at the detection limit.

Table S3. Distribution ratios, R_d, determined in the sorption experiments for the ^{14}C-CEM III/C + $CaCO_3$ binary system.

Initial activity, ($Bq·mL^{-1}$)	S/L, ($g·L^{-1}$)	Time, (days)	R_d, ($L\ kg^{-1}$)	Error[a]	Experiment purpose
20	1	2	1184	47	Sorption kinetics (variation of S/L ratio)
20	1	7	2971	69	
20	1	14	3484	441	
20	1	30	3857	74	
20	1	60	4046	70	
20	5	2	1081	31	
20	5	7	3058	84	
20	5	14	3003	135	
20	5	30	1958	19	
20	5	60	1927	24	
20	10	2	1365	19	
20	10	7	2862	54	
20	10	14	2457	58	
20	10	30	3413	220	
20	10	60	3106	149	
5	1	2	2086	46	Sorption isotherm (variation of ^{14}C initial activity)
7.5	1	2	1884	125	
10	1	2	3154	772	
20	1	2	1896	10	
30	1	2	2698	253	
100	1	2	2404	497	
200	1	2	2679	79	
5	1	14	2793	338	
7.5	1	14	3082	56	

10	1	14	2608	160	
20	1	14	3108	233	
30	1	14	3173	88	
100	1	14	4128	87	
200	1	14	3670	37	
5	1	60	4048	138	
7.5	1	60	3303	182	
10	1	60	3384	243	
20	1	60	3095	69	
30	1	60	3614	95	
100	1	60	4910	208	
200	1	60	4544	50	
5	1	120	3165	250	
7.5	1	120	3342	72	
10	1	120	3360	53	
20	1	120	2941	60	
30	1	120	3122	60	
100	1	120	5469	1051	
200	1	120	5022	84	

[a] Uncertainties are reported as two times the standard deviation (2σ) of mean values.
[b] Measured value below the detection limit.

2.2.2. Impact of ISA: cement-^{14}C-ISA ternary system

Table S4. Distribution ratios, R_d, determined in the sorption experiments for the CEM III/C-^{14}C-ISA system carried out with $[ISA]_{tot} = 10^{-5}$-0.1 M.

Initial activity, ($Bq \cdot mL^{-1}$)	S/L, ($g \cdot L^{-1}$)	Time, (days)	$[ISA]_{tot}$, (M)	R_d, ($L\ kg^{-1}$)	Error[a]	Experiment purpose
100	10	2	10^{-5}	10766	358	Sorption kinetics (variation of ISA concentration)
100	10	2	10^{-3}	31050	3456	
100	10	2	10^{-2}	10514	882	
100	10	2	10^{-1}	1708	25	
100	10	14	10^{-5}	26093	1296	
100	10	14	10^{-3}	37485	13003	
100	10	14	10^{-2}	7930	228	
100	10	14	10^{-1}	2887	80	
100	10	60	10^{-5}	34782	6804	
100	10	60	10^{-3}	15732	429	
100	10	60	10^{-2}	25795	4161	
100	10	60	10^{-1}	5608	771	
100	10	120	10^{-5}	37789	2706	
100	10	120	10^{-3}	32712	1885	
100	10	120	10^{-2}	26988	1245	
100	10	120	10^{-1}	5411	41	

[a] Uncertainties are reported as two times the standard deviation (2σ) of mean values.

Table S5. Distribution ratios, R_d, determined in the sorption experiments for the CEM III/C + $CaCO_3$ - ^{14}C-ISA system carried out with $[ISA]_{tot} = 10^{-5}$-0.1 M.

Initial activity, ($Bq \cdot mL^{-1}$)	S/L, ($g \cdot L^{-1}$)	Time, (days)	$[ISA]_{tot}$ (M)	R_d, ($L\ kg^{-1}$)	Error[a]	Experiment purpose
100	10	2	10^{-5}	1752	37	Sorption kinetics (variation of ISA concentration)
100	10	2	10^{-3}	2420	90	
100	10	2	10^{-2}	1371	62	
100	10	2	10^{-1}	519	14	
100	10	14	10^{-5}	3168	79	
100	10	14	10^{-3}	1038	11	
100	10	14	10^{-2}	1738	69	
100	10	14	10^{-1}	861	3.2	
100	10	60	10^{-5}	2564	53	
100	10	60	10^{-3}	2372	30	

100	10	60	10^{-2}	3253	73	
100	10	60	10^{-1}	933	22	
100	10	120	10^{-5}	2500	38	
100	10	120	10^{-3}	1569	15	
100	10	120	10^{-2}	2503	16	
100	10	120	10^{-1}	1080	0.68	

[a] Uncertainties are reported as two times the standard deviation (2σ) of mean values.

2.2.3. Impact of NaCl: cement-NaCl-^{14}C ternary system

Table S6. Distribution ratios, R_d, determined in the sorption experiments for the CEM III/C – NaCl - ^{14}C system carried out with $[NaCl]_{tot} = 2.2·10^{-3}$-2 M.

Initial activity, ($Bq·mL^{-1}$)	S/L, ($g·L^{-1}$)	Time, (days)	$[NaCl]_{tot}$ (M)	R_d, ($L\ kg^{-1}$)	Error[a]	Experiment purpose
20	1	2	$2.2\ 10^{-3}$	5081	78	Sorption kinetics (variation of NaCl concentration)
20	1	2	10^{-1}	1164	26	
20	1	2	0.5	1112	93	
20	1	2	1	2639	52	
20	1	2	2	679	32	
20	1	14	$2.2\ 10^{-3}$	5432	82	
20	1	14	10^{-1}	1822	441	
20	1	14	0.5	5211	153	
20	1	14	1	6157	102	
20	1	14	2	2394	30	
20	1	60	$2.2\ 10^{-3}$	9440	360	
20	1	60	10^{-1}	5346	139	
20	1	60	0.5	24161	1132	
20	1	60	1	8704	881	
20	1	60	2	3070	199	

[a] Uncertainties are reported as two times the standard deviation (2σ) of mean values.

Table S7. Distribution ratios, R_d, determined in the sorption experiments for the CEM III/C+$CaCO_3$ – NaCl - ^{14}C system carried out with $[NaCl]_{tot} = 3.6·10^{-3}$-2 M.

Initial activity, ($Bq·mL^{-1}$)	S/L, ($g·L^{-1}$)	Time, (days)	$[NaCl]_{tot}$ (M)	R_d, ($L\ kg^{-1}$)	Error[a]	Experiment purpose
20	1	2	$3.6\ 10^{-3}$	1453	64	Sorption kinetics (variation of NaCl concentration)
20	1	2	10^{-1}	917	30	
20	1	2	0.5	824	11	
20	1	2	1	1084	65	
20	1	2	2	675	12	

20	1	14	3.6 10^{-3}	2672	74	
20	1	14	10^{-1}	1483	72	
20	1	14	0.5	2831	327	
20	1	14	1	2216	32	
20	1	14	2	1419	99	
20	1	60	3.6 10^{-3}	2464	112	
20	1	60	10^{-1}	3261	157	
20	1	60	0.5	3223	14	
20	1	60	1	1989	126	
20	1	60	2	1961	98	

[a] Uncertainties are reported as two times the standard deviation (2σ) of mean values.

2.2.4. Impact of NaCl+ISA: cement-NaCl-^{14}C-ISA

Table S8. Distribution ratios, R_d, determined in the sorption experiments for the CEM III/C – NaCl - ^{14}C system carried out with $[NaCl]_{tot}$ = 2.2·10^{-3}-2 M and constant ISA concentration ($[ISA]_{tot}$ = 10^{-2}M).

Initial activity, (Bq·mL^{-1})	S/L, (g·L^{-1})	Time, (days)	$[NaCl]_{tot}$ (M)	$[ISA]_{tot}$, (M)	R_d, (L kg^{-1})	Error[a]	Experiment purpose
100	5	10	2.2 10^{-3}	10^{-2}	7455	260	Sorption kinetics (variation of NaCl concentration in the presence of ISA)
100	5	10	10^{-1}	10^{-2}	6282	67	
100	5	10	0.5	10^{-2}	3563	81	
100	5	10	1	10^{-2}	4100	107	
100	5	10	2	10^{-2}	1164	39	
100	5	30	2.2 10^{-3}	10^{-2}	16543	448	
100	5	30	10^{-1}	10^{-2}	6641	75	
100	5	30	0.5	10^{-2}	8910	222	
100	5	30	1	10^{-2}	7272	57	
100	5	30	2	10^{-2}	2012	17	
100	5	60	2.2 10^{-3}	10^{-2}	28327	2684	
100	5	60	10^{-1}	10^{-2}	18353	946	
100	5	60	0.5	10^{-2}	14087	480	
100	5	60	1	10^{-2}	7865	208	
100	5	60	2	10^{-2}	2712	217	

[a] Uncertainties are reported as two times the standard deviation (2σ) of mean values.

Table S9. Distribution ratios, R_d, determined in the sorption experiments for the CEM III/C+$CaCO_3$ – NaCl - ^{14}C system carried out with $[NaCl]_{tot}$ = 3.6·10^{-3}-2 M and constant ISA concentration ($[ISA]_{tot}$ = 10^{-2}M).

Initial activity, (Bq·mL^{-1})	S/L, (g·L^{-1})	Time, (days)	$[NaCl]_{tot}$ (M)	$[ISA]_{tot}$, (M)	R_d, (L kg^{-1})	Error[a]	Experiment purpose
100	5	10	3.6 10^{-3}	10^{-2}	1313	17	Sorption kinetics (variation of NaCl concentration in the presence of ISA)
100	5	10	10^{-1}	10^{-2}	1678	28	
100	5	10	0.5	10^{-2}	1223	46	
100	5	10	1	10^{-2}	1230	10	
100	5	10	2	10^{-2}	846	27	
100	5	30	3.6 10^{-3}	10^{-2}	2401	27	
100	5	30	10^{-1}	10^{-2}	1821	55	
100	5	30	0.5	10^{-2}	1432	8	
100	5	30	1	10^{-2}	1354	33	
100	5	30	2	10^{-2}	838	5	
100	5	60	3.6 10^{-3}	10^{-2}	2461	40	
100	5	60	10^{-1}	10^{-2}	2000	8	
100	5	60	0.5	10^{-2}	1726	44	
100	5	60	1	10^{-2}	1360	26	
100	5	60	2	10^{-2}	1120	23	
100	5	120	3.6 10^{-3}	10^{-2}	2443	43	
100	5	120	10^{-1}	10^{-2}	2353	72	
100	5	120	0.5	10^{-2}	1702	77	
100	5	120	1	10^{-2}	1622	35	
100	5	120	2	10^{-2}	1078	18	

[a] Uncertainties are reported as two times the standard deviation (2σ) of mean values.

3. References

1. B. Lothenbach, P. Durdzinski and K. De Weerdt, *A practical guide to microstructural analysis of cementitious materials*, 2016, **1**, 177-211.
2. Y. Jo, N. Çevirim-Papaioannou, K. Franke, M. Fuss, M. Pedersen, B. Lothenbach, B. de Blochouse, M. Altmaier and X. Gaona, *Cement and Concrete Research*, 2023, **172**, 107233.
3. B. Lothenbach, G. Le Saout, E. Gallucci and K. Scrivener, *Cement and Concrete Research*, 2008, **38**, 848-860.
4. B. Lothenbach, D. A. Kulik, T. Matschei, M. Balonis, L. Baquerizo, B. Dilnesa, G. D. Miron and R. J. Myers, *Cement and Concrete Research*, 2019, **115**, 472-506.
5. E. Bernard, W. J. Zucha, B. Lothenbach and U. Mäder, *Cement and Concrete Research*, 2022, **152**, 106674.
6. G. D. Miron, D. A. Kulik and B. Lothenbach, Heidelberg, Germany, 2021.
7. M. Ochs, D. Mallants and L. Wang, *Radionuclide and metal sorption on cement and concrete*, Springer, 2016.
8. J. Tits and E. Wieland, *Radionuclide Retention in the Cementitious Near-field of a Repository for Low- and Intermediate-level Waste: Development of the Cement Sorption Database*, Nagra, National Cooperative for the Disposal of Radioactive Waste, 2023.
